# Optimization and kinetic modeling of ciprofloxacin adsorption and photocatalytic degradation in water

**DOI:** 10.1038/s41598-025-29266-x

**Published:** 2025-12-08

**Authors:** Hanan Taha, Amal Zaher, Nabila Shehata

**Affiliations:** Environmental Science and Industrial Development Department, Faculty of Postgraduate Studies for Advanced Sciences, Beni-Suef University, Beni-Suef, Egypt

**Keywords:** Chemistry, Environmental sciences, Materials science

## Abstract

The persistent presence of pharmaceutical residues in aquatic environments presents a significant ecological and public health challenge, necessitating innovative and sustainable remediation strategies. This study investigates the dual functionality of a synthesized nickel–aluminum layered double hydroxide (Ni–Al LDH) composite for the efficient removal of ciprofloxacin (CIP), a widely used antibiotic, from wastewater through both adsorption and photocatalytic degradation processes. The Ni–Al LDH was synthesized via co-precipitation and characterized using FE-SEM, EDX, XRD, FTIR, XPS, TGA, and BET techniques. Adsorption experiments demonstrated a maximum adsorption capacity of 14.03 mg g^− 1^ under optimized conditions, including an initial CIP concentration of 20 mg L^− 1^, a contact time of 5 min, an adsorbent dosage of 0.125 g L^− 1^, and a pH of 11. The photocatalytic degradation under solar irradiation achieved a removal efficiency of 78.7% at 60 min using 0.02 g of catalyst and an initial CIP concentration of 15 mg L^− 1^. The kinetics and adsorption isotherm modeling confirmed the material’s high reactivity and affinity toward CIP. The novelty of this work lies in the application of dual-function Ni–Al LDH for the removal of a pharmaceutical pollutant, demonstrating rapid adsorption kinetics and an enhanced photocatalytic activity, a solar-driven, and a green approach involving ethanol and plant-based reagents for material regeneration, aligning with the sustainable water treatment goals. These findings highlight the material’s promise as an eco-friendly and efficient candidate for practical wastewater treatment applications.

**Supplementary Information:**

The online version contains supplementary material available at 10.1038/s41598-025-29266-x.

## Introduction

Water is one of the main pillars of the ecosystem; it’s important for drinking, agriculture, industry, and other uses. However, there is an extensive increase in water pollution caused by organic or non-organic pollutants such as agricultural pesticides, industrial, chemicals (like heavy metals^1^, and dyes^2^, oils, nutrients (phosphates and ammonia), pharmaceutical residues (antibiotics and synthetic hormones^3^, and biological pollution (pathogens whether they are viral or bacterial infections). Hence, they mainly affect the water through humans and animal feces, because of lacking a strong sewage system, which can lead to severe water pollution. Antibiotics have been used on a wide scale for humans and animals as a treatment for some bacterial infections, to pertain to their health, thus the remaining amounts of the residues in one way or another got released to the aquatic life, causing severe damage to the ecosystem and the living biota^4^. Lately, they have been detected in many other sources of water like groundwater, sewage effluent, and hospital effluent, and then discharged into the water system^5^. In recent years, the widespread use of antibiotics in the medical field has led to an extensive increase in antibiotic-resistant bacterial infections in the ecosystem, leading to a massive threat to the prevention and treatment of wastewater^6^, and the development of severe risks to human health.

Ciprofloxacin (CIP) is considered as one of the most potent antibiotics. Currently, it is used for the treatment in both human medicine and livestock, since it affects a wide variety of microorganisms^7^. CIP is a second-generation member of the quinolone antibiotic group, while highly efficient, it also exhibits low biodegradability^7^, and may contribute to the development of antibacterial resistance genes if not properly treated from the ecosystem. CIP has been reported to affect the nervous system, blood circulation, and potentially cause heart toxicity. Therefore, it is essential to find a simple, low-cost, effective, and applicable route for CIP removal from wastewater. Currently, various technologies are employed for the removal of CIP from wastewater, including physical, chemical, and biological techniques such as membrane separation^8^, ion exchange reaction^9^, coagulation^10^, biodegradation^11^, photocatalysis^12^, and adsorption^13^. However, the adsorption and photocatalysis methods appear to be the most convenient, affordable, effective, simple to design, and insensitive to toxic pollutants. Adsorption has gained growing interest due to its simplicity, high efficiency, and broad applicability. The effectiveness of this method primarily depends on the choice of adsorbent material. Moreover, photocatalysis is known as a sustainable green technology, where it mainly utilizes solar energy^14^. It helps improve the degradation of antibiotics in wastewater^15^.

For instance, the integration of hydroxyapatite (HA) and TiO₂ has proven effective, achieving complete degradation and mineralization of CIP under UV light​^16^. Europium decorated titanium dioxide/poly(vinylidene fluoride) Eu–TiO₂/PVDF membranes, another promising material, were shown to adsorb and photodegrade CIP under visible light, indicating their potential for treating mixed pollutants^17^. Additionally, carbon nanotubes functionalized with hydroxyl and carboxyl groups offer high adsorption capacity, while hybrid systems combining magnetite with advanced oxidation processes further enhance CIP degradation, demonstrating practical and cost-effective solutions for water treatment​. Recent studies have highlighted various Layered double hydroxide (LDH)- based hybrid materials as efficient adsorbents and photocatalysts for CIP removal from wastewater. Layered double hydroxides and their derived layered double oxides (LDOs) remain highly attractive nanomaterials for the adsorption of organic and agricultural pollutants^18,19^. Structurally resembling hydrotalcite clays, LDHs consist of positively charged layers formed by divalent and trivalent metal cations arranged in octahedral coordination. The interlayer region, known as the interlamellar space, typically contains carbonate anions, which can be replaced or modified with other desired anions to tailor the material’s functionality. Recent advancements have underscored the versatility of LDHs and their composites, showing their applicability in areas such as photocatalysis, energy storage, nanocomposite fabrication, and water treatment. Due to their notable anion exchange capability, LDHs are widely utilized for the selective removal of anionic contaminants, including dyes, antibiotics, and pharmaceutical residues^20,21^. Moreover, the addition of another element with a high absorption rate can enhance the process of adsorption^22,23^. Non-noble metal catalysts such as cobalt (Co), copper (Cu), magnesium (Mg), nickel (Ni), iron (Fe), aluminum (Al), and zirconium (Zr) have attracted considerable attention due to their wide availability and cost-effectiveness^24,25^. Although previous studies have examined LDHs for either adsorption or photocatalysis, very few studies have explored their dual functionality for both processes, especially under solar irradiation conditions.

The novelty of this study lies in the synthesis and dual-functional application of Ni-Al LDH material that effectively serves as an adsorbent and a photocatalyst for the removal of CIP. Distinct from conventional studies that primarily rely on artificial ultraviolet (UV) irradiation, the present work uses natural sunlight as a more sustainable and energy-efficient technique, which enhances environmental compatibility and reduces operational costs and energy consumption. To increase the removal efficiency of CIP, process parameters (pH, adsorbent dose, initial concentration and time) were optimized and regeneration & recyclability, besides cost analysis were studied. Kinetic and isothermal modeling were performed, providing a clearer understanding of the material’s behavior and efficiency. The practical relevance of this work lies in its potential application in treating effluents from hospitals and pharmaceutical industries, where antibiotic residues are frequently detected. By leveraging solar-driven photocatalysis and efficient adsorption, this approach could be seamlessly integrated into existing water treatment infrastructures to improve the removal of persistent drug residues, thereby contributing to safer water management and environmental protection.

## Materials and methods

### Materials

Aluminum chloride LR (Anhydrous) AlCl_3_ with 98% purity, was purchased from ALPHA CHEMIKA (India), Nickel (II) Chloride hexahydrate NiCl_2_.6H_2_O, and sodium hydroxide (NaOH) were both obtained from PIOCHEM for laboratory chemicals in EGYPT with 98% purity. CIP was obtained from PHARCO Pharmaceuticals, EGYPT. Pure methanol (98%) and deionized water have been used throughout the whole experiment.

### Preparation of LDH

As showing in Fig S1, Ni-Al LDH was synthesized using the coprecipitation method with a Ni: Al molar ratio of 4:1. First, NiCl₂·6 H₂O and AlCl₃ were accurately weighed and dissolved in distilled water under continuous stirring to form a homogeneous aqueous solution. Then, NaOH solution (2 M) gradually added dropwise under vigorous stirring until pH reached 10 ± 0.1. The resulting mixture was agitated for 24 h at room temperature. Afterward, the suspension was centrifuged, and the solid residue was washed three times with distilled water until pH of the washings reached 7. Finally, the obtained product was dried in a vacuum oven at 60 °C for 24 h and then ground into a fine powder to yield Ni-Al LDH^26^.

### Characterization

The morphological structure and composition of Ni-Al LDH, both prior to and after the adsorption, and the photocatalytic degradation processes were obtained by Scanning electron microscopy –Energy dispersive spectrometer, (ThermoFisher, USA) Quattro S Felid Emission Gun. The crystallographic structure of the materials was recorded by X-ray diffraction pattern (Brucker 2D Phaser 2nd generation, Germany). Using the XRD technique with CuKα radiations (λ = 1.54060 Å) and 30 KV and 10 mA. The scans were recorded in the 2θ range 10–80° with a step size of 0.01° (0.5 s/step). Fourier transform infrared spectroscopy (FT-IR, VERTEX 70 Bruker Optics, Germany) was used to record the FT-IR spectra of Ni-Al LDH before and after the photocatalytic degradation and adsorption processes. The chemical states and elemental composition of the samples were qualitatively analyzed using X-ray Photoelectron Spectroscopy (XPS) which collected on K-ALPHA (Thermo Fisher Scientific, USA) with monochromatic X-ray Al K-alpha radiation-10 to 1350 eV, spot size 400 μm at pressure 10 − 9 mbar, with full spectrum pass energy 200 eV, and at narrow spectrum 50 eV. The analysis was performed with an Al Kα radiation source (hν = 1486.6 eV), operated at a voltage of 12.5 kV. Thermogravimetric analyses (TGA/DTG) were reported by (Stream Instrumentation Manufacturer, France). The specific surface area of the materials was obtained by N_2_ adsorption-desorption isotherm which was conducted at 77 K using (Belsorp-minix, Japan) using Brunauer–Emmett–Teller (BET) method.

### Adsorption arrays

For optimizing the adsorption capacity of Ni-Al LDH towards the CIP, optimization studies were carried out, using a rotary orbital shaker (Heidolph Unimax 1010, Germany) at 200 rpm with varied doses of LDH (0.02 to 0.125 g). The pH was adjusted using 0.1 M HCl or NaOH solutions, measured with a digital pH meter (Jenway 3510, UK) with values of 3, 5, 7, 9, and 11, and initial conc. ranged from 1 to 50 mg L^− 1^ to identify the optimum dose, pH, and initial concentration, respectively. Equilibrium time was identified using two concentrations of CIP (20 and 40 mg L^− 1^) at times ranging from 0 to 300 min. Samples were collected at regular intervals and analyzed via UV–VIS spectrophotometry at 275 nm. The reusability test was conducted at pH 11, dose 0.125 g and 20 mg L^− 1^ of CIP solution using ethanol (solvent) and green tea as a green reagent.

### Photocatalytic degradation arrays

To investigate the photocatalytic degradation of CIP onto Ni-Al LDH applying sunlight energy, a series of parameter optimization studies was carried out at a latitude of 29° 03’ 60.00” N, and a longitude of 31° 04’ 60.00” E, at Beni-Suef University (Beni Suef, Egypt). All the experiments were conducted at 12 pm to 5 pm under sunlight during the fall season, using a rotary orbital shaker (Heidolph Unimax 1010, Germany) at 200 rpm. A range of doses of Ni-Al LDH (0.05 to 0.125 g) was used, and the pH was adjusted using 0.1 M HCl or NaOH solutions, measured with a digital pH meter (Jenway 3510, UK), while the initial concentration was measured at 1, 3, 5, 10, 15, 20, 25, 30 and 50 mg L^− 1^ while keeping the other factors constant for the impact of dose, pH and initial concentration constant, respectively. To identify the equilibrium time, the time varied from 0 to 300 min using two concentrations of CIP (20 and 40 mg L^− 1^). Samples were collected at regular intervals after their exposure to the sunlight and analyzed via UV–VIS spectrophotometry at 275 nm. The reusability test was carried out at pH 11, a dose of 0.5 g, and 20 mg L^− 1^ of the CIP solution using ethanol and green tea as solvents.

### Process kinetics

The kinetics studies were conducted using five common models; Pseudo-first order (P1O), Pseudo-second order (P2O), Avrami, mixed 1st and 2nd order (M12O), and intraparticle diffusion models. The P1O model considers that the quantity of vacant adsorption sites corresponds directly with the adsorption rate (Eq. 1). It is often applied for systems with physisorption processes or where adsorption occurs on a homogeneous surface^27^. The P2O model assumes the adsorption rate is calculated as the square of the number of empty sites (Eq. 2). This model is widely applied for chemisorption processes where chemical bonds are formed between adsorbate and adsorbent^28^. The M12O is particularly useful for describing adsorption processes where neither P1O nor P2O kinetics alone provide an accurate fit, often due to complex interactions between adsorbate and adsorbent. The model suggests that adsorption could initially follow P1O kinetics at low concentrations but may transition to P2O behavior as the process progresses (Eq. 3), possibly due to changes in surface binding sites or the formation of multilayers^28^. Avrami model (Eq. 4) is well-suited for adsorption systems with multiple stages or complex kinetics^29^. For adsorption kinetics, Avrami model was adapted from its initial application in crystallization studies^30^. The intraparticle diffusion (IPD) model (Eq. 5) considers that adsorption is regulated by the adsorbate particles’ diffusion into the porous structure of the adsorbent, often applied to multilayer adsorption^31^.

1 $${{\text{q}}_{{\text{t =}}}}{{\text{q}}_{\text{e}}}\left( {1 - {{\text{e}}^{ - \,{\text{k}}}}{{_{1}}^{\text{t}}}} \right)$$

2 $$\:{q}_{t}=\frac{{k}_{2}{q}_{e}^{2}\:t}{{1+q}_{e}{k}_{2\:}*t}$$

3 $$\:\text{q}\text{t}\:=\:\text{q}\text{e}\frac{1-\text{e}\text{x}\text{p}(-kt)}{1-{f}_{2\:}\text{e}\text{x}\text{p}(-kt)}$$

4 $${{\text{q}}_{\text{t}}}={\text{ }}{{\text{q}}_{\text{e}}}[1\, - \,{\text{exp}}{\left( { - \,{{\text{k}}_{{\text{av}}}}{\text{t}}} \right)^{{\text{na}}}}^{{\text{v}}}]$$

5 $${{\text{q}}_{\text{t}}}={\text{ }}{{\text{k}}_{{\text{id}}}}{\text{ }}{{\text{t}}^{1/2}}+{\text{ C}}$$

Where q_t_ is the amount of adsorbate adsorbed at time t (mg g^− 1^), q_e_​ is the amount of adsorbate adsorbed at equilibrium (mg g^− 1^), K_1_​ is P1O rate constant (min^− 1^), K_2_​ is P2O rate constant (g mg^− 1^ min^− 1^), ƒ is the coefficient (dimensions) of M12O model, k is the adsorption rate constant of M12O model (mg^− 1^ min^− 1^), k_avt_ is Avrami rate is constant (dimensionless), n_av_ is the Avrami exponent (dimensionless), K_id_ is the intraparticle diffusion rate constant (mg g^− 1^ min^− 1/2^), and C is a constant related to the boundary layer thickness (mg g^− 1/2^).

For photocatalytic degradation, P1O(deg.) and P2O(deg.) were applied according to Eqs. 6 and 7, respectively^32^.

6 $${\text{Ln }}\left( {{{\text{C}}_{\text{o}}}{\text{/}}{{\text{C}}_{\text{t}}}} \right)\,=\,{{\text{K}}_{{\text{obs1}}}}{\text{t}}$$

7 $$\:\frac{1}{Ct}=\:\frac{1}{{C}_{o}}+\:{k}_{obs2}\:t$$

Where C_o_ and C_t_ represent CIP concentrations at time 0 and t, respectively and k_obs1_ and k_obs2_ refer to the observed rate coefficients of P1O and P2O, respectively. The rate constants and the corresponding correlation coefficients have been determined from the plots of Ln (C_o_/C_t_) vs. t and 1/C_t_ vs. t for P1O and P2O models, respectively.

### Adsorption isotherm modeling

To further understand the adsorption mechanism, different models were investigated. Langmuir isotherm (Eq. 8) assumes monolayer adsorption on a homogeneous surface with identical sites, in which the adsorbed molecules do not interact^33^. Adsorption on heterogeneous surfaces is described by Freundlich isotherm (Eq. 9), often suitable for multilayer adsorption^34^. Dubinin-Radushkevich isotherm (Eq. 10) was used to estimate the mechanism of adsorption in microporous materials, useful for distinguishing between physical and chemical adsorption^35^. Brouers-Sotolongo (Baudue) isotherm (Eq. 12) integrates features of Freundlich and Langmuir models and it is applied to explain adsorption on heterogeneous or fractal-like surfaces. It provides flexibility in fitting data with complex surface heterogeneities, making it suitable for materials with non-uniform pore structures^36^. Khan isotherm (Eq. 13) is useful for adsorption systems involving both homogeneous and heterogeneous adsorption processes. This model is particularly useful for complicated systems since the adsorbate interacts with multiple types of active sites, offering a more comprehensive approach than traditional isotherms^37^.

8 $$\:\text{q}_\text{e}\:=\:\text{q}_{\text{m}\text{a}\text{x}}\frac{{k}_{L}c}{1+{k}_{L}c}\:$$

9 $${{\text{q}}_{\text{e}}}={\text{ }}{{\text{K}}_{\text{f}}}{{\text{C}}_{\text{e}}}^{{1/{\text{n}}}}$$

10 $${{\text{q}}_{\text{e}}}{\text{= }}{{\text{q}}_{{\text{max }}}}{\text{exp}}\left( { - \,{{\text{k}}_{{\text{ad}}}}{\upvarepsilon^2}} \right)$$

11 $$\:\varepsilon\:=\text{R}\text{T}\:\text{l}\text{n}\left[1+\frac{1}{{C}_{e}}\right]$$

12 $$\:\text{q}\text{e}=\:\frac{{q}_{m}{b}_{o}{C}_{e}^{1+x+y}}{1+{b}_{o}{C}_{e}^{1+x}}$$

13 $${{\text{q}}_{{\text{e =}}}}\left( {{{\text{q}}_{\text{m}}}{{\text{b}}_{\text{k}}}{{\text{c}}_{\text{e}}}} \right){\text{/ }}{\left( {{\text{1}}\,{\text{+}}\,{{\text{b}}_{\text{k}}}{{\text{c}}_{\text{e}}}} \right)^{{\text{ak}}}}$$

Where q_max_ is the maximum adsorption capacity (mg g^− 1^), K_L_​ is Langmuir constant related to the affinity of the binding sites (L mg^− 1^), C_e_ is the equilibrium concentration of adsorbate in solution (mg L^− 1^), K_f_ is Freundlich constant and it is indicative of adsorption capacity (mg g^− 1^)·(L mg^− 1^)^1/n^, 1/n is a heterogeneity factor, n is the adsorption intensity (dimensionless), k_ad_ is D-R constant related to the mean free energy of adsorption (mol^2^ kJ^− 2^), ε is the Polany potential, R is the gas constant (8.314 J mol·K^− 1^), T is the temperature (K). K​ is Khan isotherm constant (L mg^− 1^), a_k_​ is Khan isotherm constant related to the equilibrium (L mg^− 1^)^n^, and b_k_ is Khan constant.

## The results and discussion

### Characterization of the materials

#### SEM and EDX

**Fig. 1:**
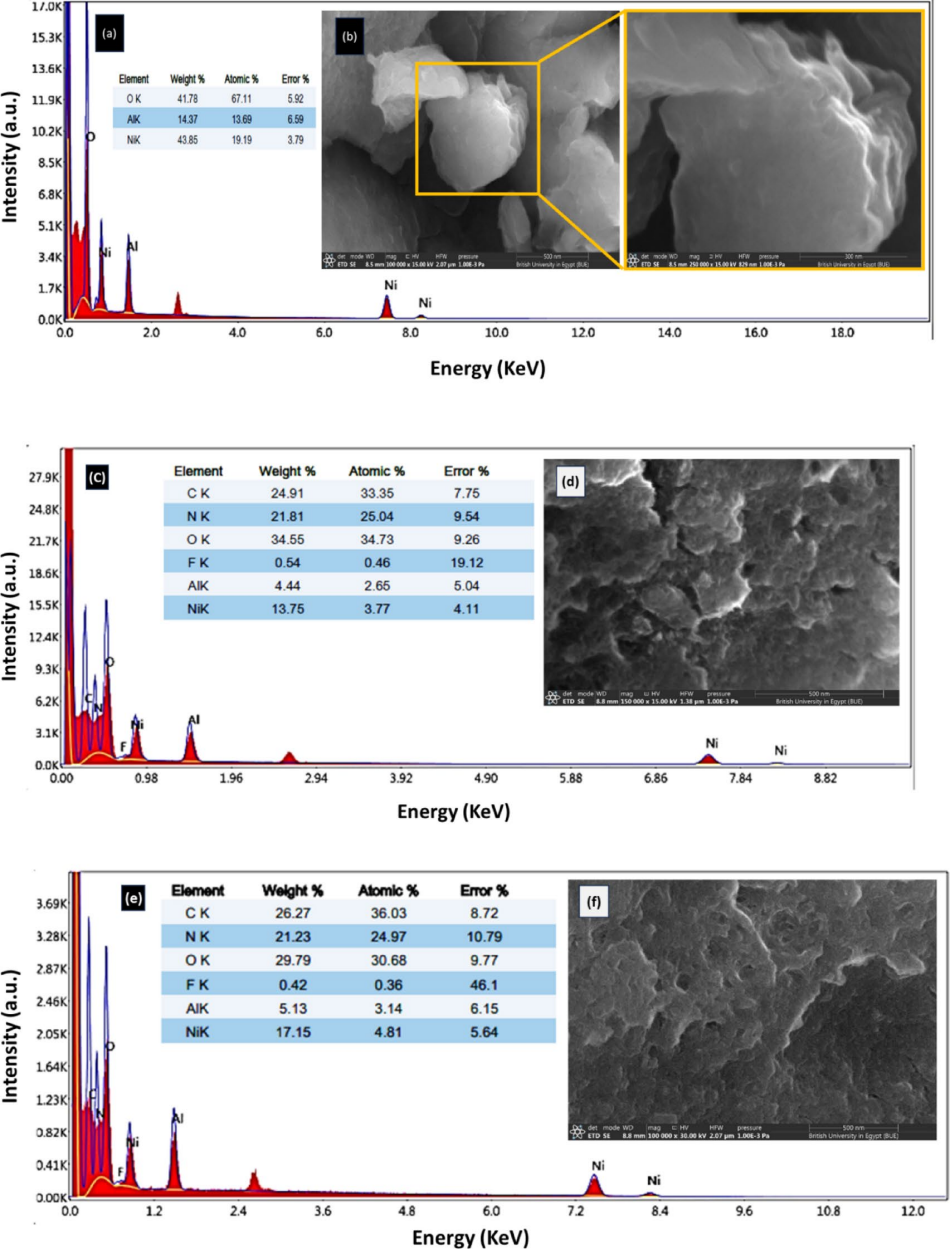
**(a)** EDX analysis of Ni-Al LDH (onset is its SEM image **(b)**), **(c)** and **(d)** EDX and SEM image of CIP after adsorption onto LDH, respectively, **(e)** and **(f)** EDX and SEM image of the photocatalytic degradation of CIP by LDH.

The EDX spectra for the pristine Ni-Al LDH (Fig. 1a) shows dominant peaks corresponding to Ni and Al. These peaks verify the existence of Ni and Al, which are the primary constituents of the LDH. As expected, Ni (43.85%) is present in larger amounts than Al (14.37%), which aligns with the stoichiometry of the LDH. No peaks from external elements or impurities are present, confirming that the pristine LDH sample is clean and pure. The SEM image of the pristine LDH sample (Fig. 1b) exhibited a highly layered, sheet-like structure, with distinct edges and folds. The layers were relatively smooth and more structured, reflecting the typical morphology of LDHs. After CIP adsorption, the EDX spectra (Fig. 1c) shows notable changes in the elemental composition. The peaks for Ni and Al are still present, but some additional peaks as well as changes in intensity can be observed. There is a rise in signals which is explained by the existence of carbon. This indicates that CIP, an organic compound rich in carbon, has been successfully adsorbed onto the Ni-Al LDH surface. The presence of CIP molecules slightly masks the Ni and Al signals. After CIP adsorption (Fig. 1d), the SEM revealed a more aggregated and less defined structure. The sheets and layers became more compact due to CIP molecules occupying surface and interlayer spaces. The roughness of the surface increased significantly, indicating strong interactions between CIP and Ni-Al LDH. This rough and agglomerated texture pointed to successful adsorption. However, the image doesn’t confirm whether the adsorption of CIP onto the LDH is of a chemical or physical nature. After photocatalytic degradation (Fig. 1e), there are new signals referring to C (26.27%), N (21.23%) and F (0.42%) which confirm the existence of CIP. Following the photocatalytic degradation of CIP (Fig. 1f), the surface morphology appears to have undergone further degradation. The structure becomes rough and irregular, like the post-adsorption image, but the degradation process seems to have caused additional changes along with holes and cracks in the surface of the catalyst. Notably, the surface now has more pores or voids, which could be attributed to restoring some of the material’s surface area, which suggests the occurrence of photodegradation.

#### XRD

**Fig. 2:**
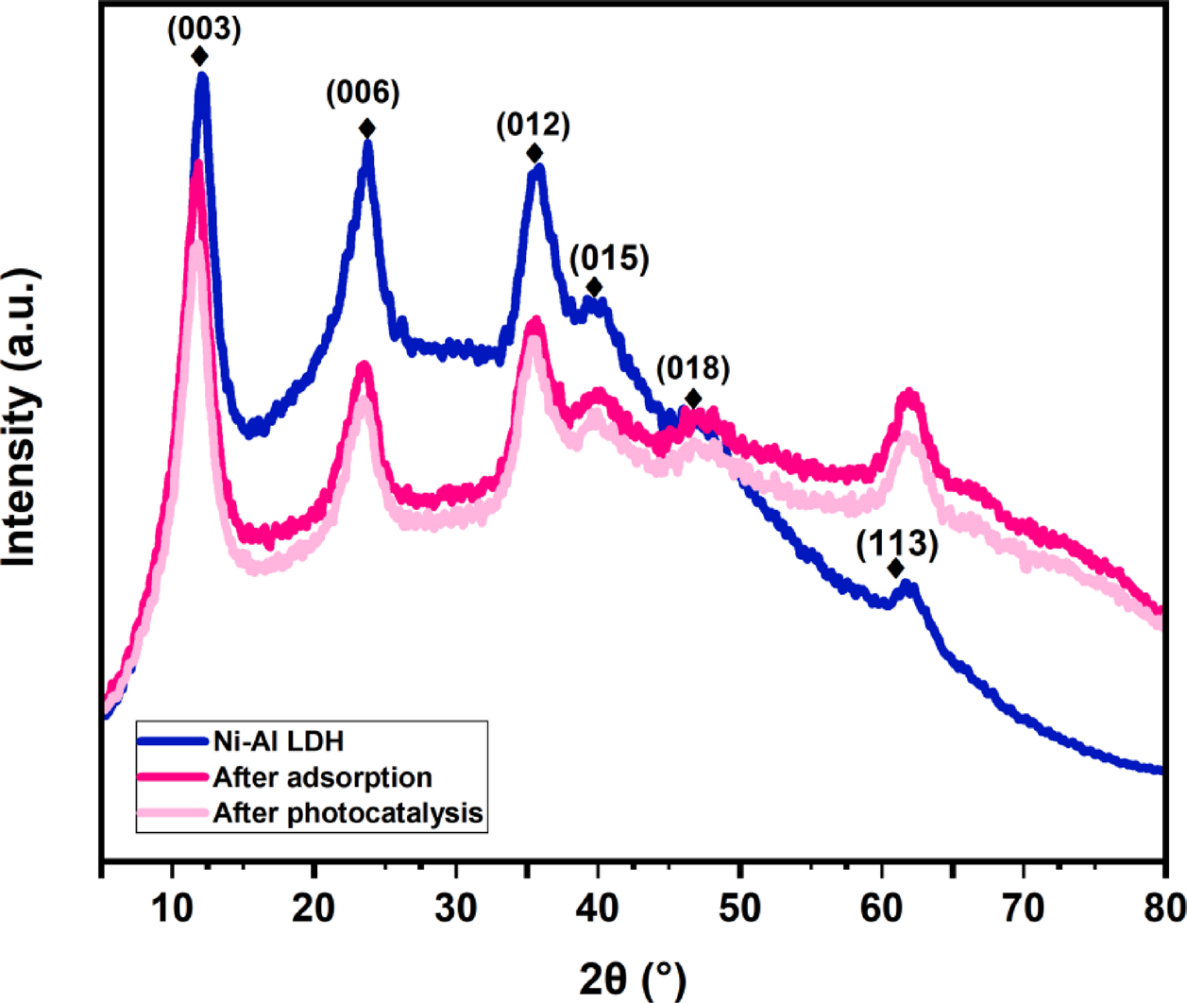
Ni-Al LDH XRD patterns prior to and following adsorption and photocatalytic degradation of CIP.

The crystalline structure of the produced Ni-Al LDH and the effect of CIP intercalation on the material was examined using X-ray diffraction (XRD) analysis. The XRD pattern (Fig. 2) demonstrates sharp and well-defined peaks, characteristic of the layered double hydroxide (LDH) structure. These peaks indicate high crystallinity with typical basal spacing. The positions and intensities of the peaks verify the Ni-Al LDH’s layers structure with characteristic diffraction peaks at 2θ 11.74°, 23.47°, 34.67°, 39.38°, 46.74°, and 61.16°, corresponding to the (003), (006), (012), (015), (018), and (113), indicating a well-defined layered structure with a high degree of crystallinity that was synthesized under co-precipitation method, and match well with the previous studies^5,38,39^. There are no other peaks that show the crystalline and extremely pure LDH layers. These peaks agree with standard patterns for LDH materials (JCPDS PDF 00–056-0953) and (JCPDS PDF 00–022-0452). After adsorption of CIP onto Ni-Al LDH, the pattern retains most of the characteristic peaks of the pristine LDH but shows a slight broadening of the peaks, a shift in basal spacing (003​) from 11.7˚ to 11.1˚ and reduction in intensity of the (006) and (012). These changes suggest structural alterations, likely due to CIP molecules intercalating into the LDH layers or adsorption onto the surface. After photocatalytic degradation of CIP, the XRD pattern also exhibits broader and less intense peaks compared to that of LDH, a shift in basal spacing (003) from 11.7˚ to 10.7˚ suggesting partial loss of crystallinity. However, as unexpectedly, the LDH remains with high crystallinity after the photodegradation process suggesting good chemical strength of the materials after treatment. This is agreed with SEM results.

#### FT-IR

**Fig. 3:**
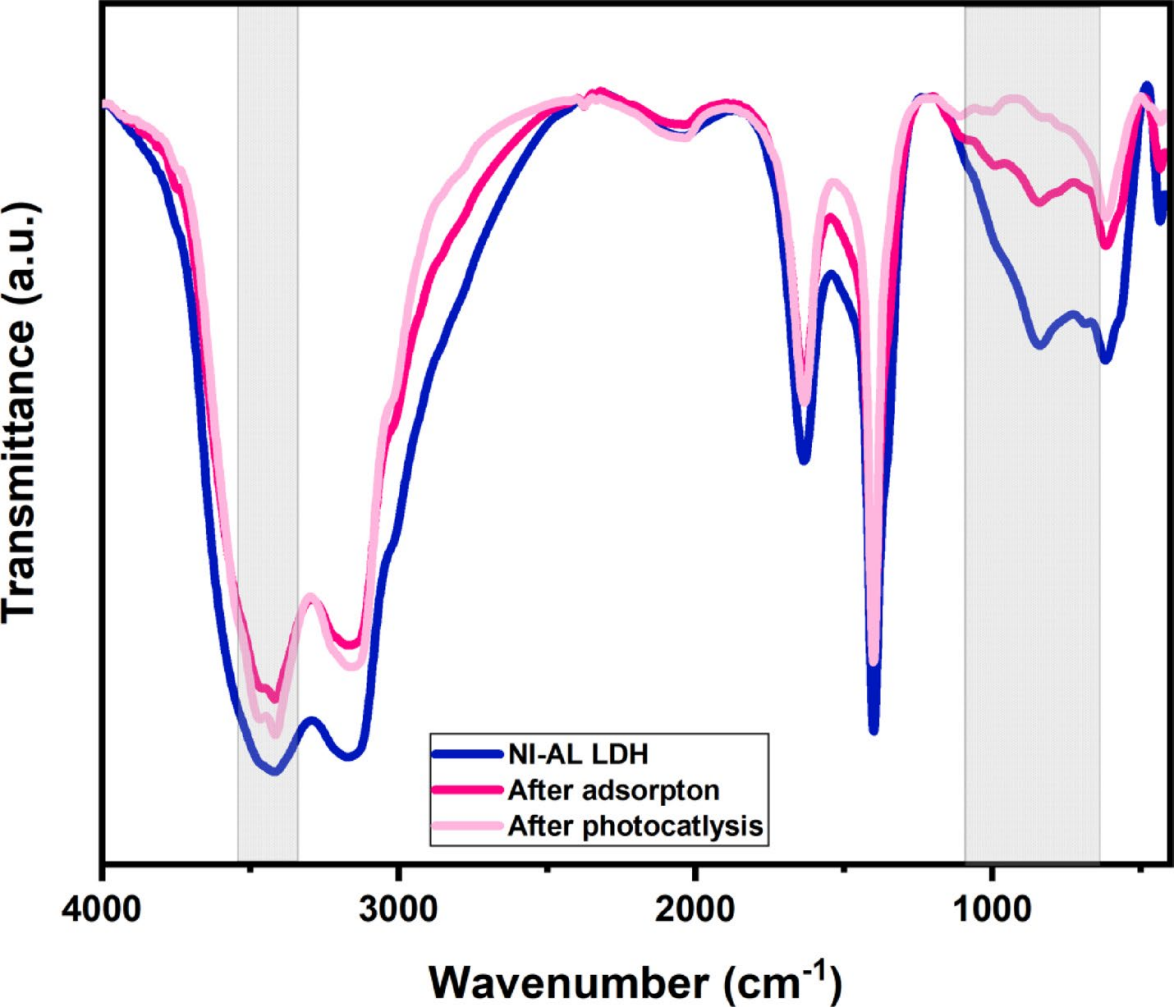
FT-IR patterns of Ni-Al LDH prior to and following adsorption and photocatalytic degradation of CIP.

The FT-IR spectrum of Ni-Al LDH (Fig. 3), shows high-intensity bands at 3418.681 cm⁻¹ and 3170.887 cm⁻¹ representing the O–H stretching vibrations due to the presence of the hydroxyl groups in the Ni-Al LDH^40^, while the bands at 2038.048 cm⁻¹, 1633.765 cm⁻¹ correspond to the bending vibrations of the H_2_O molecules present in the hydrotalcite structure. The bands around 1350–1500 cm⁻¹ are typically associated with interlayer carbonate anions in the LDH. The bands 839.758 cm⁻¹, 618.255 cm⁻¹, and 433.353 cm⁻¹^41^, represent the metal oxide bending bands, confirming the existence of Ni–O or Al–O in the structure of Ni-Al LDH^42^. After the adsorption of CIP onto Ni-Al LDH (Fig. 3), a decrease in the peak’s intensity related to O–H stretching vibrations at 3812.524 cm⁻¹, 3743.338 cm⁻¹, compared to Ni-Al LDH, because of the interaction between the adsorbed CIP and the OH groups present in the LDH through a hydrogen bond. The peak at 3418.681 cm⁻¹ was shifted to 3417.040 cm⁻¹ and it also shows a splitting which may be attributed to the existence of the molecules of CIP with functional groups like, C = O and N–H stretching bonds onto the LDH surface. At 1632.654 cm⁻¹ compared to the original sample (1633.765 cm⁻¹), the H_2_O bending vibrations show a slight decrease, proving that H_2_O molecules are present, and could be displaced or interacted with the CIP molecules. The peak of metal hydroxyl at 1399.225 cm⁻¹, indicates a slight reduction in intensity, even though, the peak is still strong, proving that after the adsorption of CIP molecules, the LDH core is retained. A minor peak formed at 990.513 cm⁻¹ showing the possible interaction among LDH and the adsorbed CIP molecules^43^. After the photocatalytic degradation of the CIP onto LDH. The broad O-H stretching peaks of the hydroxyl group region (3600–3200 cm⁻¹) show reduced intensity, indicating the consumption of hydroxyl groups during the production of reactive oxygen species (ROS) like hydroxyl radicals^44^. In the carbonyl and carboxyl region (1700.000–1600.000 cm⁻¹), the reduction of the C = O stretching peak confirms the degradation of CIP carboxyl groups, consistent with mineralization into CO₂ and H₂O^45^. The aromatic C = C stretching region (1600.000–1400.000 cm⁻¹) also shows decreased peak intensity, indicating the oxidative breakdown of the CIP aromatic ring^44^. Finally, in metal-hydroxyl (M-OH) and the metal-oxygen (M-O) region (800.000–500.000 cm⁻¹), shifts or intensity changes suggest structural modifications in the LDH due to redox reactions involving the metal centers during photocatalysis^46^.

#### XPS

**Fig. 4:**
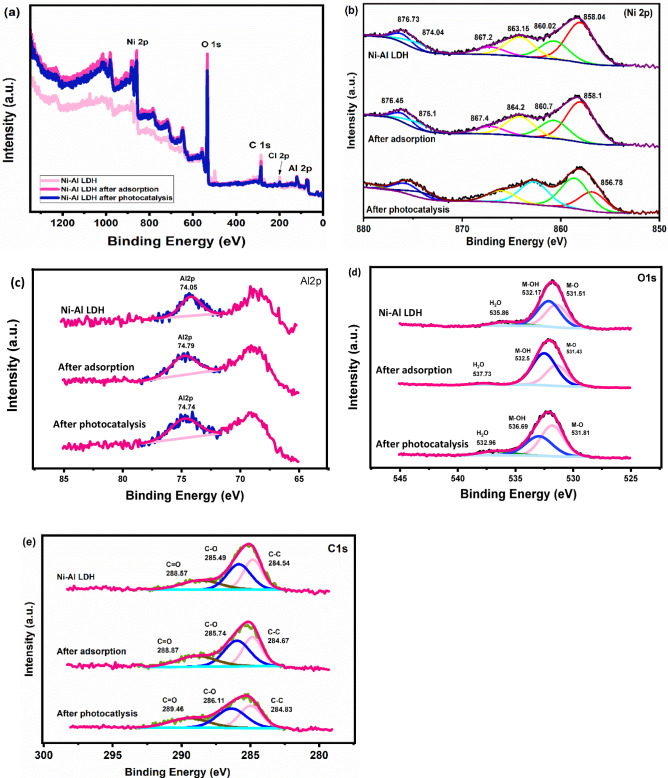
Ni-Al LDH XPS patterns prior to and following adsorption and photocatalytic degradation of CIP: **(a)** full survey, **(b)** Ni 2p, **(c)** Al 2p, **(d)** O 1 s and **(e)** C 1s.

XPS analysis was conducted to investigate the chemical composition and surface interactions of the Ni–Al LDH before and after CIP adsorption and photocatalytic degradation. The analysis confirms the presence of the primary constituent elements Ni, Al, O, and C in all three samples (Fig. 4a). The corresponding atomic percentages are C 1 s (44.07%), O 1 s (34.99%), Al 2p (15.52%), and Ni 2p (5.42%). In the pristine sample, the main Ni 2p₃/₂ peak is located at 858.04 eV, with multiple satellite peaks observed at 860.02, 863.15, 867.16, 874.04, 874.68, and 876.07 eV (Fig. 4b). These features are characteristic of Ni²⁺ in an octahedral environment with associated shake-up satellites, confirming the presence of divalent nickel in the LDH framework. The atomic percentage of Ni at this stage is 5.42%, indicating a moderate presence of nickel on the surface. After adsorption, the Ni 2p₃/₂ peak slightly shifts to 858.1 eV, while the satellite peaks appear at 860.7, 864.2, 867.4, 875.1, and 876.45 eV. This is accompanied by a slight increase in Ni atomic percentage to 5.76%, suggesting enhanced surface exposure or interaction of Ni with adsorbed species. Following photocatalysis, the Ni 2p₃/₂ peak remains at 856.78 eV, with satellite features persisting at similar binding energies, and the atomic percentage slightly decreases to 5.64%. The consistency in binding energy and the presence of satellite structures across all stages confirm that Ni remains in the + 2-oxidation state throughout, without undergoing any redox transformation. However, the subtle shifts in satellite peaks and atomic percentage indicate that Ni sites are involved in surface-level interactions and possibly contribute to the catalytic activity through coordination with intermediates or surface restructuring. Overall, while Ni maintains electronic stability, the XPS data support its active role in surface processes during both adsorption and photocatalytic reactions^47,48^. The Al 2p peak in Ni-Al LDH is observed at 74.05 eV (Fig. 4c), with an atomic percentage of 15.52%, which corresponds to Al³⁺ in a stable octahedral coordination within the LDH structure. Upon adsorption, the Al 2p binding energy shifts upward to 74.79 eV, accompanied by a substantial increase in atomic percentage to 32.55%, indicating that Al sites become more surface-exposed and experience a more electron-deficient environment, likely due to structural reorganization or stronger coordination with adsorbed species. Following photocatalysis, the Al 2p peak slightly shifts to 74.74 eV, and the atomic percentage remains elevated at 32.35%, suggesting that Al maintains its oxidized state but continues to participate in surface-level interactions^49,50^. The O 1 s spectrum of Ni–Al LDH exhibited dominant peaks at 531.51 eV, 532.17 eV, and 535.86 eV (Fig. 4d), corresponding to M-O, M-OH, and H_2_O, respectively, which are key adsorption sites for CIP. After CIP adsorption, a notable increase in the intensity of the M-OH at 532.5 eV peak was observed, suggesting the presence of oxygenated organic moieties from CIP interacting with the LDH surface. Following photocatalysis, a mild increase in M-O at 531.81 eV, and M-OH at 536.69 was detected, consistent with degradation of the organic layer and possible restoration of active sites. Initially, the H₂O-related peak was low, indicating minimal surface moisture. After CIP adsorption, it increased to 537.73 eV, due to enhanced hydrogen bonding with CIP’s polar groups. Following photocatalysis, the intensity slightly decreased to 532.96 eV, likely from water consumption during •OH radical formation and desorption under light exposure, reflecting dynamic surface changes. Atomic % rose from 34.99% to 39.38% after adsorption due to the contribution of CIP’s oxygen-containing functional groups and surface hydroxyl interaction, then slightly decreased to 37.58% as oxygen species were consumed or transformed during photocatalytic oxidation^51,52^. The C 1 s peaks, initially at 284.54 eV, 285.49 eV, 288.57 eV, corresponded to C-C, C-O, C = O, respectively. After the adsorption, these peaks shifted to 284.67 eV, 285.74 eV, and 288.87 eV, while after photocatalysis shifted to 284.83 eV, 286.11 eV, and 289.46 eV. The corresponding atomic % dropped significantly from 44.07% in Ni-Al LDH to 22.32% after adsorption and further to 21.80% after photocatalysis. This trend confirms successful uptake of the organic pollutants followed by their oxidative degradation, with loss of carbonaceous residues, confirming adsorption and degradation of CIP^7,53^. A new Cl 2p peak appeared at 199.54 eV after photocatalysis with an atomic % of 2.64% (Fig. 4e), likely originating from CIP degradation byproducts. These findings indicate that CIP adsorbs primarily onto surface Ni–OH and oxygen sites, altering the local chemical environment, and is effectively degraded through photocatalysis, leading to partial recovery of surface-active species and exposure of internal LDH components, thereby confirming the dual functionality of Ni–Al LDH as both adsorbent and photocatalyst.

#### TGA

**Fig. 5:**
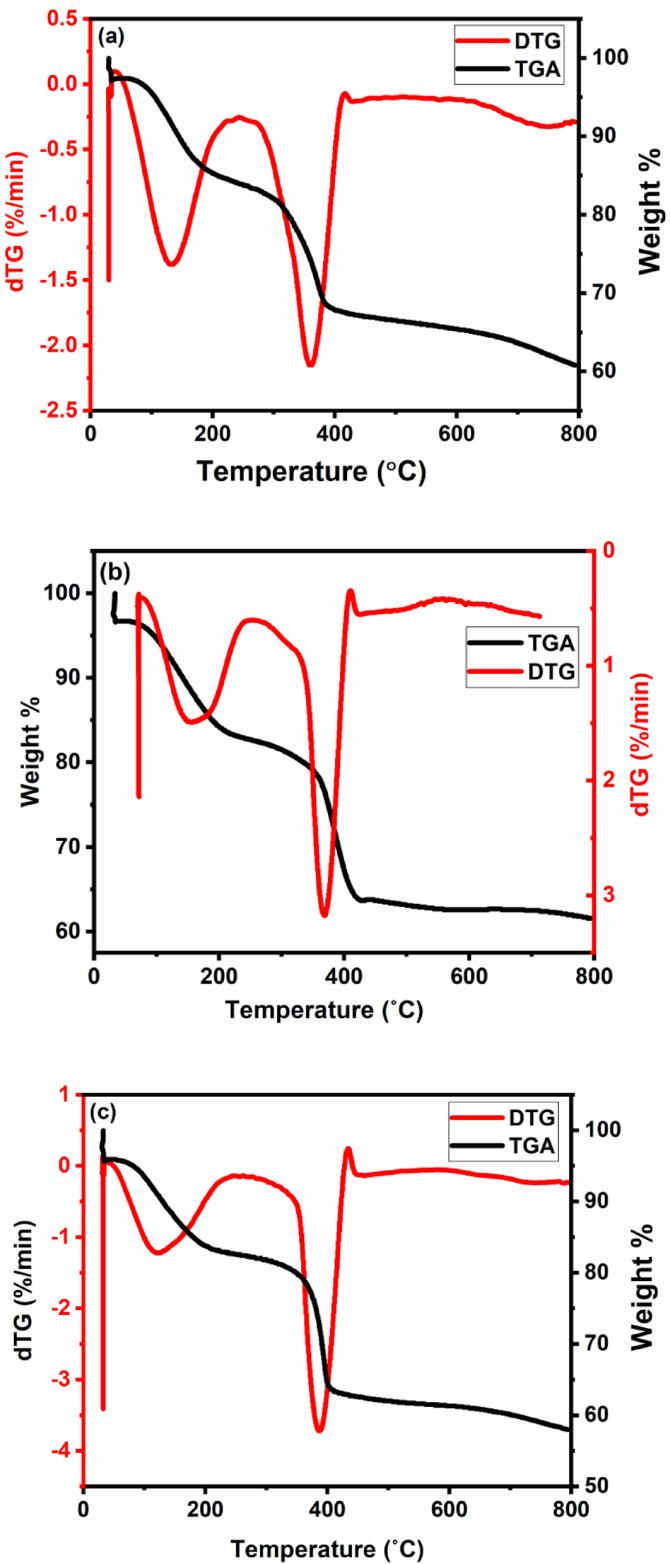
Ni-Al LDH TGA patterns prior to **(a)** and following adsorption **(b)** and photocatalytic degradation of CIP **(c)**.

The thermal evolution TGA/DTA of Ni-Al/LDH is shown on Fig. 5. The thermogravimetric analysis (TGA) of the pristine Ni–Al LDH (5a) revealed a typical three-step weight-loss pattern: the first stage, occurring at 57.5–208 °C, and a weight loss of 2.561 mg (12.45%) occurs, this corresponds to the loss of physically adsorbed moisture and interlayer water molecules weakly bound to the LDH galleries. The second stage occurred at 276.58–419.06 °C, a more significant weight loss of 3.223 mg (15.66%) was recorded, where this is associated with the initial dehydroxylation of the brucite-like layers and partial release of interlayer anions, commonly carbonate. The third stage, extending above 500 °C, represents the major structural decomposition due to complete dehydroxylation, collapse of the layered structure, and transformation into mixed Ni–Al oxide phases, after which the mass stabilizes, indicating the formation of thermally stable oxides^54,55^. In the case of Ni–Al LDH after CIP adsorption (5b), the initial dehydration step remained evident to 60.08–215.63 °C, with weight loss increasing to 3.768 mg (13.17%), suggesting higher water content. This may be due to the adsorption of CIP molecules. The most notable change was a significantly higher weight loss between 323.16 and 432.56 °C, a larger weight loss of 4.822 mg (16.85%), higher than the pristine sample. This additional loss is attributed to the pyrolytic degradation of ciprofloxacin molecules strongly adsorbed on the LDH surface and within its interlayer spaces, producing volatile products such as CO₂, NH₃, and small organics, as reported for CIP thermal decomposition. This organic decomposition process overlaps with the LDH dehydroxylation step, resulting in a larger total mass loss compared to the pristine material, thereby confirming the effective adsorption and retention of the antibiotic^56,57^. After photocatalytic degradation (5c), the TGA profile retained the dehydration stage at 48.06–209.66 °C, with a weight loss of 2.453 mg (12.50%), similar to that of the pristine sample, indicating the removal of moisture and possibly small organics that remained after catalytic degradation, but showed a markedly reduced weight loss at 352.74–436.65 °C, with a weight loss of 3.329 mg (16.96%), slightly higher than pristine but lower than the adsorbed sample^58^. This suggests that most of the CIP had been mineralized during photocatalysis, leaving only trace residual organics on the LDH surface. Across all samples, the residual mass > 500 °C was comparable, confirming that regardless of adsorption or photocatalytic treatment, the LDH ultimately transformed into thermally stable Ni–Al oxide phases. At 800 °C, residual mass was 67.46%, showing high thermal stability. After adsorption, the residual mass was 63.73% slightly lower than pristine, which reflects the extra organic load being combusted. After photocatalysis, a residual mass of 62.77% was recorded, the lowest among the three samples, possibly due to the degradation of CIP or the formation of byproducts.

#### BET

**Fig. 6:**
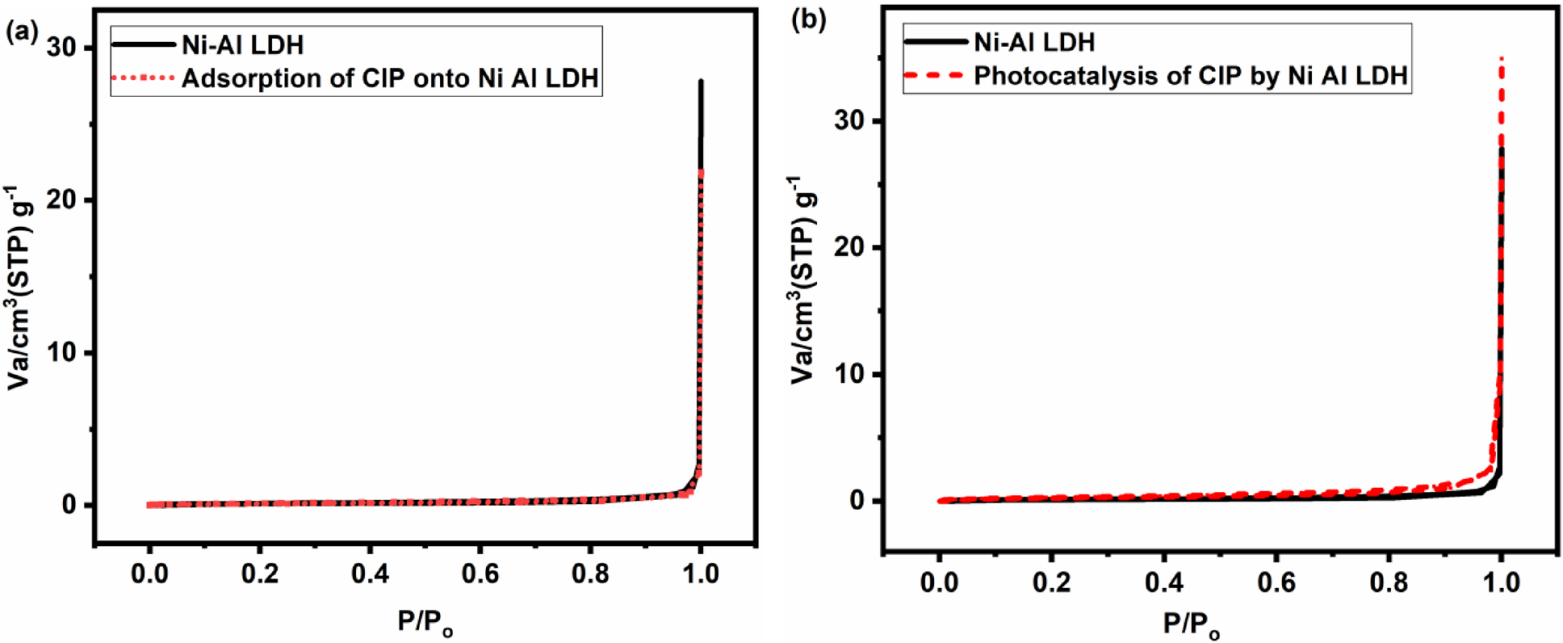
N_2_ adsorption/desorption isotherm patterns of Ni-Al LDH before and after adsorption **(a)** and photocatalytic degradation of CIP **(b)**.

The N_2_ adsorption-desorption of Ni-Al LDH prior to adsorption and photocatalysis is shown in Fig. 6. The BET surface area for Ni-Al LDH was 0.40 m^2^ g^− 1^, the pore volume of 0.09 cm^3^ STP g^− 1,^ and the average pore diameter of 28.4 nm, which indicates that the material has a relatively low surface area, and according to IUPAC standards, it has IV types with H3-type hysteresis, which is characteristic for mesoporous materials with slit-like pore shape^59^. After the adsorption of CIP, a decline in the BET surface area of 0.37 m^2^ g^− 1^ and the average pore diameter of 27.6 nm was observed. This is probably due to the surface-active sites being occupied by the CIP molecules and partially blocking the mesopores, which hinders pore accessibility^60^. On the other hand, after the photocatalytic degradation, the BET surface area rose to 0.94 m^2^ g^− 1^, with a pore volume of 0.21 cm^3^ STP g^− 1^. However, the broadening of hysteresis loops likely demonstrates an enhancement of the mesopores. This could be attributed to the additional generation of voids^61^ which resulting in an increment in the specific surface area of the material after photocatalysis.

### Optimization of the adsorption factors

#### Impact of pH

**Fig. 7:**
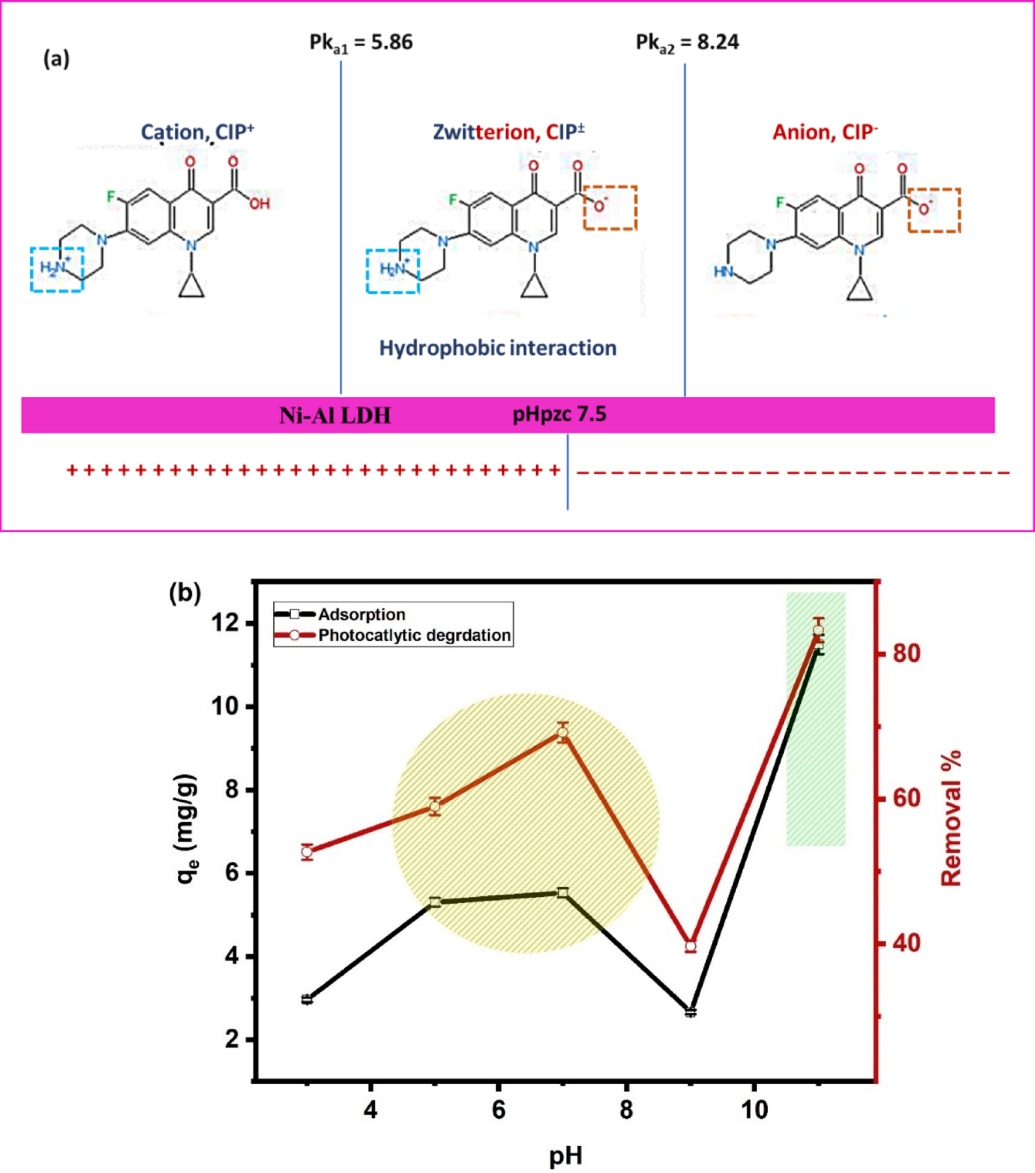
The ionization states of CIP **(a)** and impact of pH on adsorption capacity and photocatalytic degradation of CIP by Ni-Al LDH **(b)**.

CIP molecule exists in the solution in three ionization states as shown in Fig. 7a; anionic at pH > 8.24, zwitterionic at 5.86 < pH < 8.24, and cationic form at pH < 5.86^62^. As shown in Fig. 7b, as pH increased from 3 to 5 mg g^− 1^, the adsorption capacity started to increase slightly and the adsorption capacity reached its maximum value in the range of pH 5–7, followed by a drop at pH 9, then it rose significantly with an increase in pH up to 11, which was the optimum pH. As the pH at the point of zero charge (pH_pzc_) of Ni-Al LDH is 7.5 ^42^, at low pH values 3–5, the Ni-Al LDH surface becomes more positive, and the CIP molecules carry a positive charge because of the protonation of their amine groups^63^. Therefore, both CIP molecules and the Ni-Al LDH surface will carry a positive charge, leading to repulsion force interactions. This explains why the adsorption capacity of CIP molecules onto the surface of Ni-Al LDH is low in this region. Meanwhile, at pH values of 7.5, where the pH_pzc_ is close to zero, the Ni-Al LDH surface has minimal net charge, and electrostatic interactions between the CIP and the LDH surface are drastically reduced, affecting adsorption capacity. Additionally, the zwitterionic form of CIP is characterized by a hydrophobic nature, which promotes the adsorption of CIP onto Ni-Al LDH. However, at pH 11, CIP becomes more anionic, and hydroxyl groups on the LDH surface strongly increase adsorption capacity by forming hydrogen bonds with the negatively charged CIP molecules. The deprotonated carboxylic group in CIP interacts with hydroxyl groups on the Ni-Al LDH surface through hydrogen bonding. This could explain the dramatic increase in adsorption capacity^64^. As shown in Fig. 7b, the process of photocatalytic degradation exhibits a similar pH-dependent trend to adsorption, where it shows relatively high efficiency at pH 3–7, the lowest efficiency at pH 9, and a dramatic increase in degradation at pH 11, which is the optimum pH for photocatalysis. At low pH values 3–5, photocatalysis is efficient due to the formation of hydroxyl radicals during the degradation process. These radicals enhance electrostatic interactions and contribute to effective degradation, even though the repulsion between the positively charged CIP and protonated Ni-Al LDH surface^42^. At pH 7.5–9, photocatalytic efficiency drops significantly. Since the Pk_a2_ =8.24 (Fig. 7a), the molecules of CIP transformed from a zwitterionic state to ionic state, and the repulsion force increased, resulting in low interactions between the LDH surface and CIP molecules, leading to the lowest degradation efficiency in this range^64^. At pH 11, photocatalytic degradation reaches its peak efficiency. In the strongly alkaline medium, hydroxyl groups on the Ni-Al LDH surface increase, promoting hydrogen bonding with the deprotonated carboxylic group of CIP molecules. These interactions, combined with the enhanced production of hydroxyl radicals in alkaline conditions, lead to the highest degradation efficiency at this pH^38^.

#### Impact of dose

**Fig. 8:**
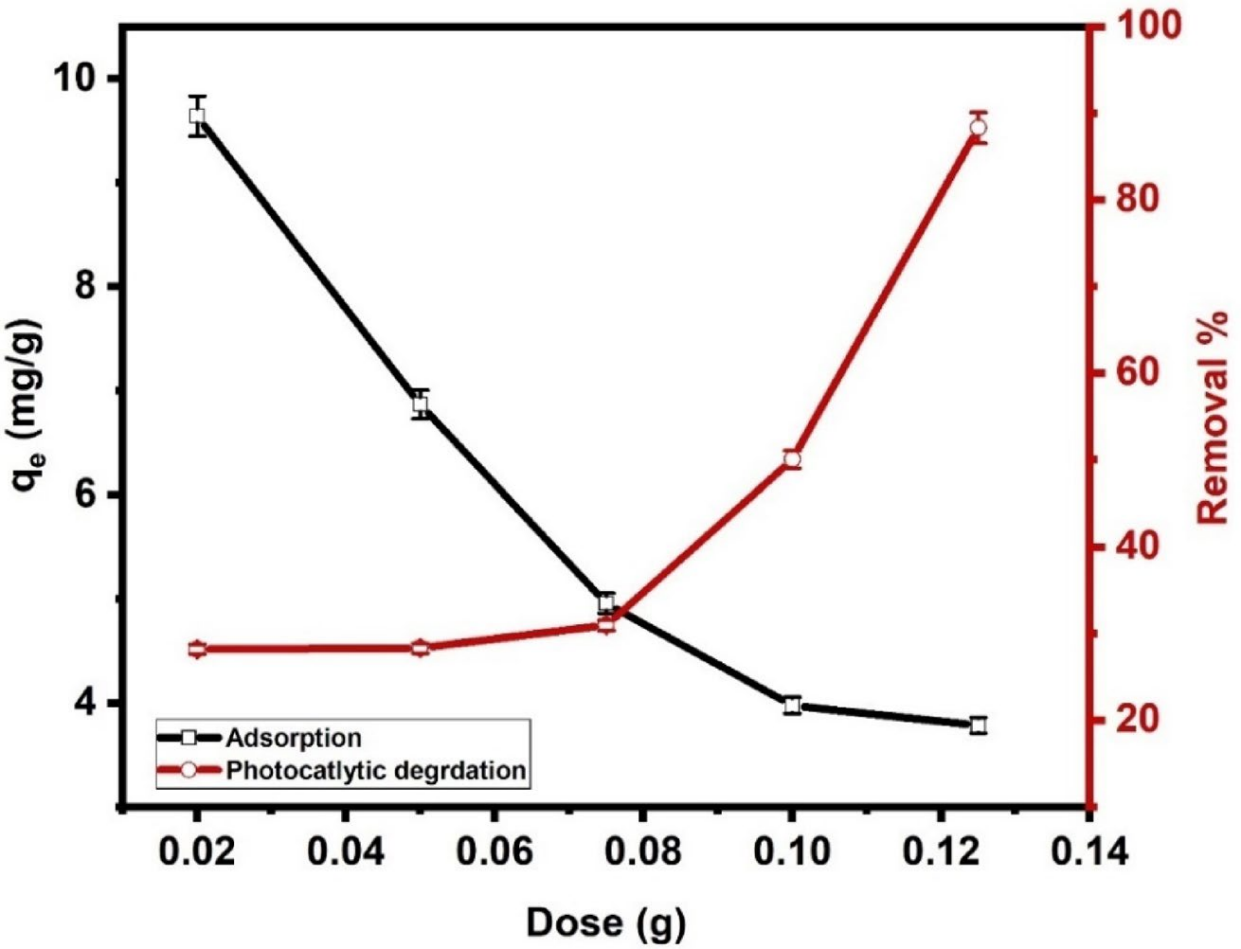
Impact of Ni-Al LDH dose onto the adsorption and photocatalytic degradation of CIP by Ni-Al LDH at pH 11, and initial concentration of 20 mg L^− 1^.

Figure 8 indicates that as the dose of Ni-Al LDH rises from 0.02 to 0.125 g, the adsorption capacity (mg g^− 1^) decreases from 9.6 to 3.7 mg g^− 1^, exhibiting an inverse relationship. This trend can be explained by the concept of the “adsorbent dose effect,” where a higher Ni-Al LDH dose causes the adsorption capacity to decrease due to the unsaturation of active sites. When the Ni-Al LDH dose is low, the available active sites are fully utilized, resulting in higher adsorption capacity. However, particle aggregation may occur as the dose increases, reducing the overall surface area available for CIP molecules to attach to, thus decreasing the adsorption capacity^65^. Additionally, as more adsorbent is added, many active sites remain unoccupied due to limited CIP molecules in the solution. This reduction in efficiency with higher adsorbent doses aligns with the literature, where surface saturation and agglomeration effects are common explanations for this trend in heterogeneous adsorbent systems^66^. Figure 8 shows that photocatalytic degradation efficiency slightly increases as the Ni-Al LDH dose increases up to 0.0759 g, then significantly increases as the dose rises to 0.125 g. At low catalyst doses, the number of active sites for photon absorption is insufficient, resulting in low degradation efficiency. As the dose increases, more catalyst particles become available, enhancing photon absorption and increasing the ROS production^67^. In this study, the adsorption process shows an optimal dose of around 0.02 g with a high adsorption capacity of 9.6 mg g^− 1^, while photocatalysis appears to perform better at a higher dose of around 0.125 g with degradation percent of 88.3%. This suggests that different doses optimize each process, reflecting that adsorption relies on surface area and active sites, and photocatalysis depends on photon absorption and catalyst interaction.

#### Impact of initial concentration

**Fig. 9:**
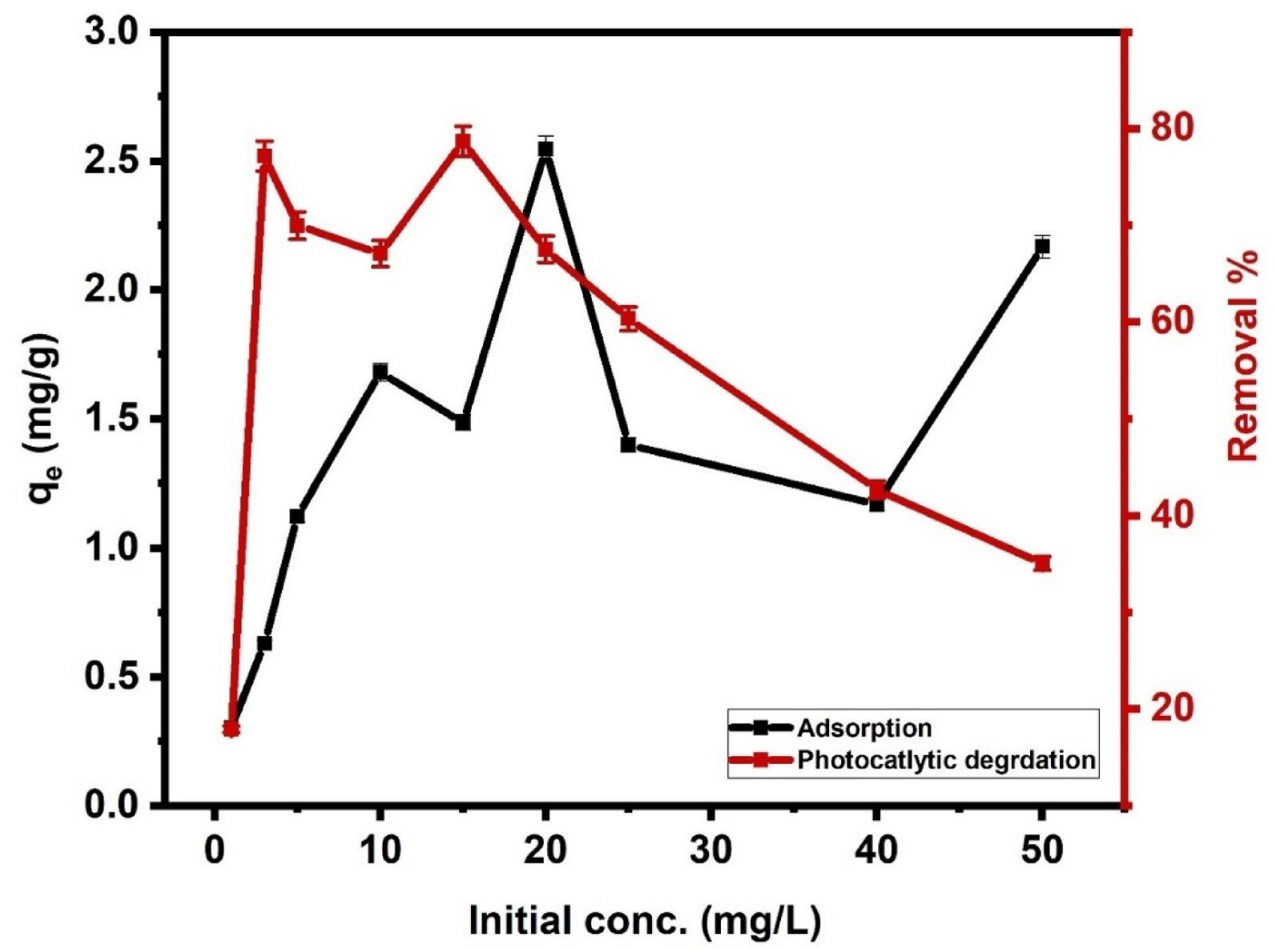
Impact of CIP initial concentration on the adsorption and photocatalytic degradation of CIP by Ni-Al LDH at pH 11.

Figure 9 indicates a progressive rise in the capacity of adsorption (q_e_) with rising CIP concentration, and the capacity reaches its maximum at initial concentration 20 mg L^− 1^. Further, an increase in the CIP initial conc doesn’t show a noticeable increase in q_e_. This suggests that the fluctuation in the adsorption capacity at concentration above 20 m mg L^− 1^ indicates that the available active sites become saturated, limiting further adsorption. This behavior is typical for adsorption processes where, after a certain concentration, the surface of the adsorbent cannot accommodate additional molecules, even with increasing adsorbate concentrations^68^. For photocatalytic degradation (Fig. 9), at lower concentrations, the degradation efficiencies were relatively high, with values of 77.19%, and 70% for 3 and 5 mg L^− 1^, respectively. This trend indicates that at lower CIP concentrations, Ni-Al LDH provides sufficient active sites for effective degradation, and light penetration remains optimal for generating ROS. The degradation efficiency exhibited a slight decline when the initial concentration rose to 10, reflecting the growing competition for active sites on the photocatalyst surface, followed by a rise and peaking at 15 mg L^− 1^ with a percentage of degradation of 78.70%. At 20 mg L^− 1^, the efficiency dropped to 67.54%, and the photocatalytic process became less effective, at higher concentrations above 20 mg L^− 1^. This decline at high concentrations may be due to the reducing activation of the photocatalyst. Also, the limited number of active sites on the Ni-Al LDH could play a role, as well as the potential formation of intermediates that may temporarily occupy active sites, reducing overall photocatalytic efficiency^69,70^. These findings assume that Ni-Al LDH as a catalyst exhibits optimal performance at initial concentration *≤* 15 mg L^− 1^.

#### Impact of contact time

**Fig. 10:**
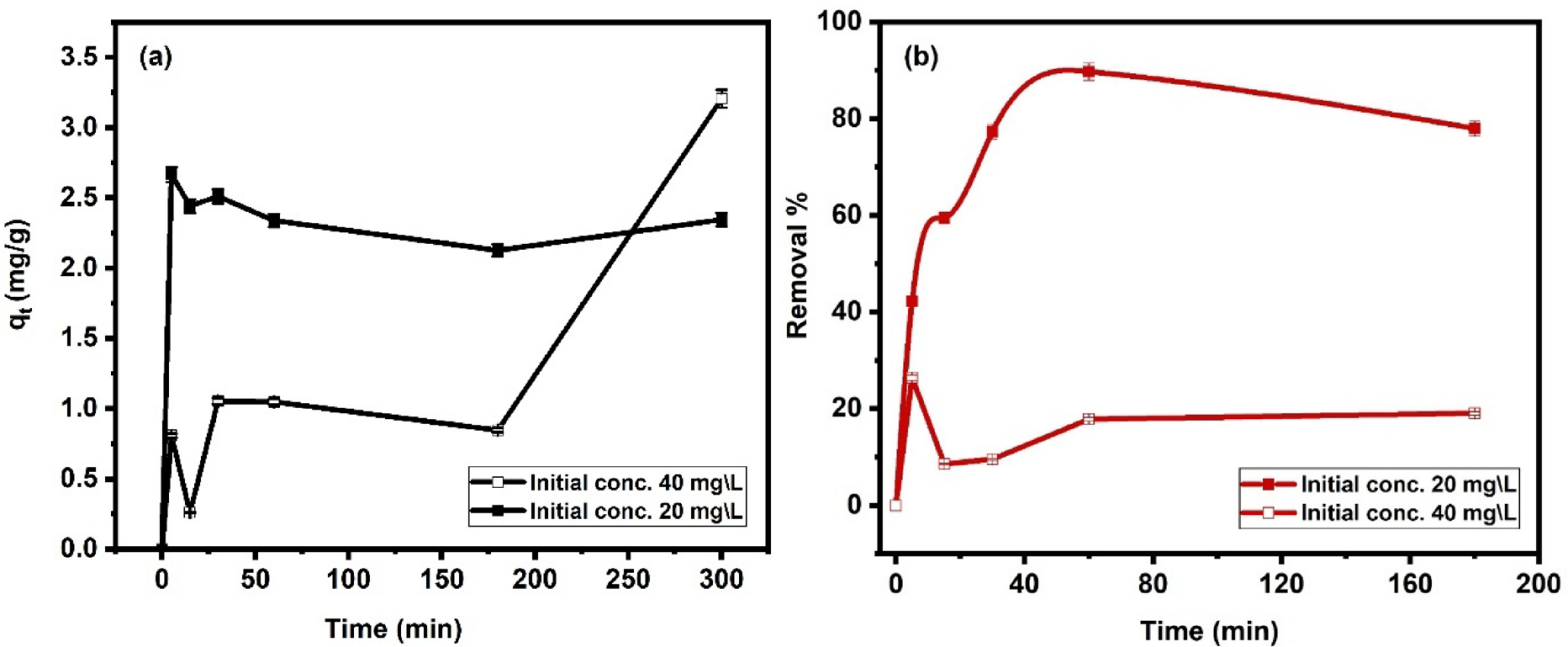
Impact of contact time on the adsorption **(a)** and photocatalytic degradation **(b)** of CIP by Ni-Al LDH at initial concentrations 20 and 40 mg L^− 1^.

The contact time plays a significant role in CIP adsorption at initial concentration 20 mg L^− 1^ (Fig. 10a) shows a rapid increase in q_t_ during the initial contact time (5 min), indicating an active adsorption process. This rapid phase is common in the adsorption process at the early stage^71^. The Ni-Al LDH surface’s accessible active sides are abundant initially, allowing for fast CIP uptake, however, increasing the time doesn’t significantly change the adsorption capacity. Surprisingly, the 20 mg L^− 1^ solution shows a higher initial q_t_ compared to 40 mg L^− 1^, suggesting that a lower concentration might favor a quicker saturation of available sites on the Ni-Al LDH surface^38^. A significant fluctuation took place, particularly in the 40 mg L^− 1^ concentration. These fluctuations may indicate possible interactions among CIP molecules in the solution, causing temporary instability in adsorption. For the initial concentration 40 mg L^− 1^, q_t_ continues to increase gradually over a more extended period (up to 350 min). This indicates a slower approach to equilibrium at higher concentrations, due to competition among CIP molecules for limited active sites on the Ni-Al LDH surface. At greater levels, the adsorption process might require longer to reach equilibrium due to higher molecular interactions and crowding on the surface of the adsorbent. This implies that the initial concentration of CIP has a significant impact on the adsorption process, which agrees with the results of the impact of initial concentration.

The impact of time on the photocatalytic degradation for CIP concentrations 20 mg L^− 1^ and 40 mg L^− 1^ is displayed in Fig. 10b. There is a rapid increase in the percentage of degradation in the early phase, indicating active photocatalytic degradation of CIP. Surprisingly, the 20 mg L^− 1^ concentration achieves a higher initial degradation percentage compared to 40 mg L^− 1^, due to the lower concentration requiring fewer active sites on the catalyst for efficient degradation. At around 30–60 min, the 20 mg L^− 1^ concentration reaches a peak of degradation percent of approximately 77%–89%, after which the degradation rate begins to decline slightly, this may be due to the depletion of CIP in the solution, causing the reaction rate to slow down as fewer molecules are available for degradation. The initial concentration 40 mg L^− 1^ shows a much lower degradation percentage, peaking at around 26.4%. This lower efficiency could be due to higher competition among CIP molecules for active sites on Ni-Al LDH, it also shows some fluctuations after the initial phase. Overextended contact times, the initial concentration 20 mg L^− 1^ shows a slight reduction in the degradation percent. In contrast, the initial concentration 40 mg L^− 1^ shows minor increases over time but does not reach the efficiency level observed at 20 mg L^− 1^. This also suggests that the initial concentration of CIP has an important influence on the photocatalytic degradation process.

### Adsorption kinetics

The modeling of adsorption and photocatalytic degradation kinetics of CIP onto Ni-Al LDH was based on several assumptions to simplify the interpretation of the experimental data. It was assumed that all experiments were conducted under constant conditions, including temperature, stirring rate, and solar irradiation intensity for photocatalysis. The adsorption process was considered to occur in a homogeneous batch system, where external mass transfer limitations were negligible due to continuous mixing. The pseudo-second-order model was employed with the assumption that chemisorption is the rate-limiting step^28^, involving electron exchange between the CIP molecules and the active sites on the LDH surface. To better capture the complexity of the reaction kinetics, the Avrami model was applied, assuming a fractional-order reaction that accounts for multi-step or diffusion-influenced behavior^29^. Additionally, the mixed 1,2 order model was used to represent systems exhibiting characteristics of both first- and second-order kinetics, particularly to describe an initial rapid uptake followed by slower equilibrium processes. All models were fitted using nonlinear regression analysis, and the selection of the most appropriate model was based on correlation coefficient (R²) values and error analysis. These assumptions provided a rational basis for interpreting kinetic behavior and evaluating the mechanism of CIP removal by the Ni-Al LDH.

**Fig. 11:**
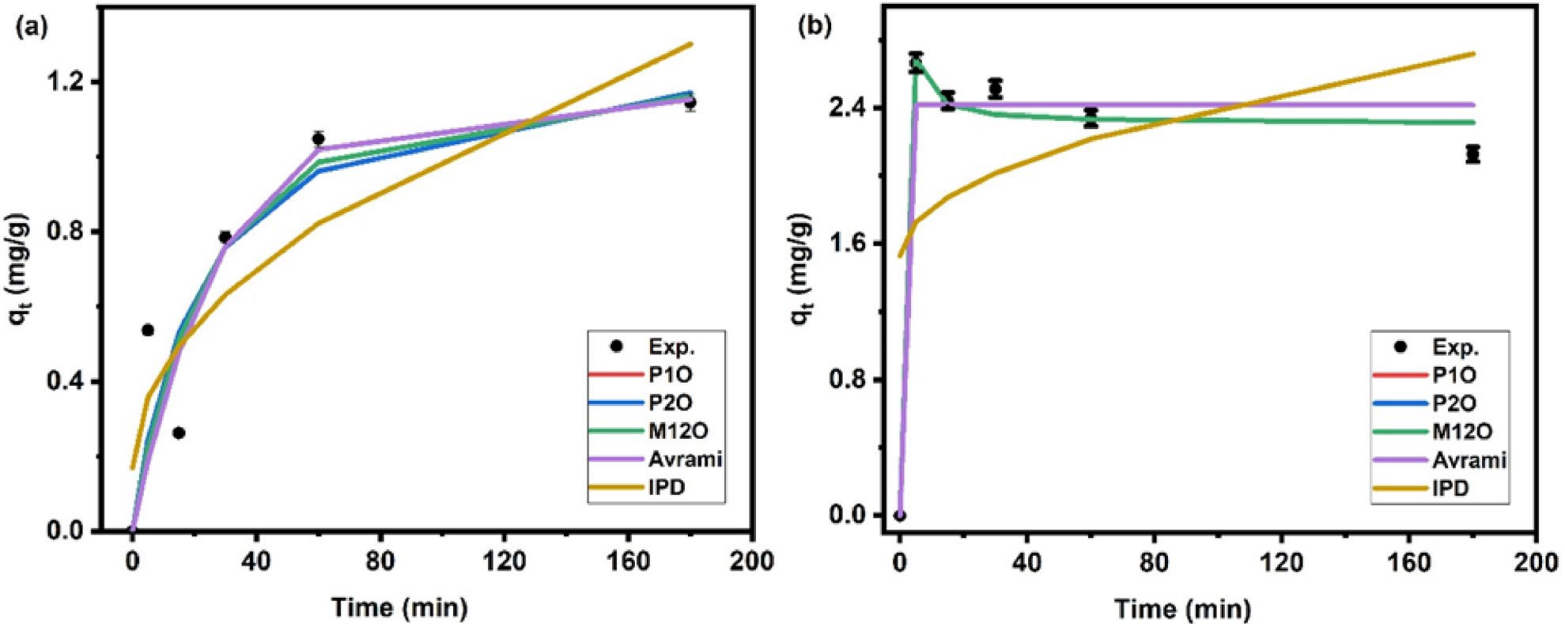
Fitting the experimental data for the CIP adsorption onto Ni-Al LDH at CIP initial concentrations 20 mg L^− 1^ **(a)**, and 40 mg L^− 1^ **(b)**.

To identify the adsorption kinetic data of CIP by Ni-Al LDH, the parameters of five models; P1O, P2O, M12O, Avrami, and IPD, were tabulated in Table 1. Figure 11a represents the fitting of five kinetic models that analyze the experimental data of CIP by Ni-Al LDH. The experimental data closely match the M12O (R^2^ = 0.833), P2O (R^2^ = 0.832), Avrmai (R^2^ = 0.831), and P1O (R^2^ = 0.830), suggesting that the physisorption (weaker, physical interactions) and chemisorption (stronger, chemical interactions) simultaneously occur during the adsorption process. This suggests potential contributions from multilayer adsorption or different kinetics at different adsorption stages^29^. While as shown in Fig. 11.b the adsorption at initial concentration 40 mg L^− 1^, equilibrium takes longer to achieve, reflecting the greater competition for adsorption sites. The M12O model fits the data best with a correlation coefficient value of R^2^ = 0.99, emphasizing that both mechanisms are still operative. The extended time to equilibrium suggests that chemisorption may play a more prominent role at higher concentrations, but physisorption still influences the initial stages^28^. In conclusion, the M12O model best describes the adsorption kinetics of CIP onto Ni-Al LDH at both concentrations, capturing the simultaneous influence of chemical and physical adsorption mechanisms. Three models; P1O, P2O, and Avrami can also fit well for this concentration with R² = 0.968. On the other hand, IPD model is poorly fit the data (R^2^ = 0.17 and 0.79 for initial concentrations 40 and 20 mg L^− 1^, respectively). Moreover, the calculated values according to this model don’t agree with the experimental one. For the kinetic of photocatalytic degradation, the results show that both P1O and P2O can control the degradation of CIP onto LDH at low concentration with high correlation coefficients; R^2^ = 0.965 and 0.982, respectively, while increasing the concentration to 40 mg L^− 1^ yield poor fitting of both models (Table 1). This indicates the kinetics are significantly influenced by the initial concentration of CIP.

**Table 1:** Adsorption kinetic models for adsorption and photocatalytic degradation of CIP by NI-AL LDH.

Models	Parameters	Adsorption		Models	Parameters	Photocatalytic degradation	
		Conc. (40 mg L^− 1^)	Conc. (20 mg L^− 1^)			Conc. (40 mg L^− 1^)	Conc. (20 mg L^− 1^)
P1O	k_1_ [min^− 1^]	3.47	0.04	P1O (deg.)	K_obs1_ [min^− 1^]	0.0015	0.0416
	q_e_ [mg g^− 1^]	2.42	1.16		R^2^	0.428	0.965
	R^2^	0.968	0.830	P2O (deg.)	K_obs2_ [min^− 1^]	6E-05	0.0911
P2O	k_2_ [g mg^− 1^ min^− 1^]	4998.15	0.035		R^2^	0.0104	0.982
	q_e_ [mg g^− 1^]	2.42	1.31				
	R^2^	0.968	0.832				
M12O	K	0.0001	0.0199				
	q_e_ [mg g^− 1^]	2.31	1.18				
	*f*_2_	1.00008	0.55				
	R^2^	0.99	0.833				
Avrami	q_e_ [mg g^− 1^]	2.42	1.16				
	k_av_	2.53	0.23				
	*n*_av_	3.28	0.16				
	R^2^	0.968	0.831				
IPD	k_ip_ [mg g^− 1^ min^− 1/2^]	0.089	0.084				
	*c*_ip_ [mg g^− 1/2^]	1.53	0.17				
	R^2^	0.17	0.79				

### Adsorption isotherm

To evaluate the capacity for adsorption, the data were fitted using numerous isotherm models; Langmuir, Freundlich, Dubinin-Radushkevich, Langmuir-Freundlich, Khan, and Baudu. The parameters of these models were listed in (Table 2).

**Fig. 12:**
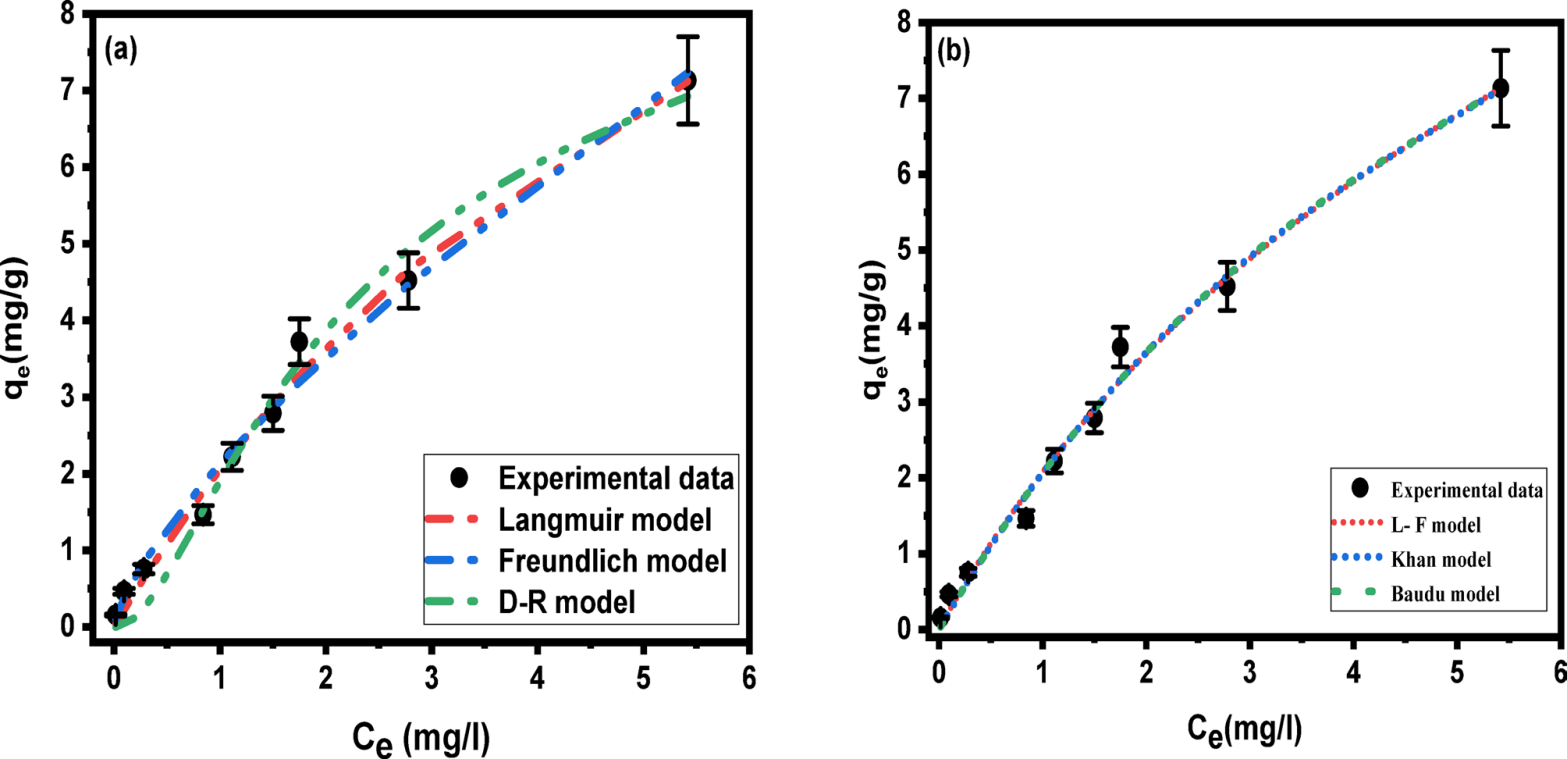
Fitting of different adsorption isotherm models to CIP onto Ni-Al LDH adsorption system.

As shown in Figure.12. Langmuir isotherm curve demonstrates a steep initial slope at low concentrations, with a correlation coefficient of 0.96 and q_m_ = 14.03 mg g^− 1^ indicating high adsorption affinity, followed by a plateau at higher concentrations, suggesting monolayer adsorption and saturation of adsorption sites. More complex models, such as Baudu shows intricate curves that reflect the influence of multiple factors such as adsorbate-adsorbate interactions and surface heterogeneity on the process of adsorption. However, the model describes the data well with R^2^ = 0.95. Freundlich isotherm curve exhibits a more gradual increase in adsorption capacity without a clear plateau with an R^2^ = 0.95, reflecting the heterogeneous nature of the Ni-Al LDH surface and multilayer adsorption. Dubinin-Radushkevich isotherm curve also gives a good fit with an R^2^ = 0.96, that showed that the process of adsorption is predominantly physical. On the other hand, hybrid models, like Langmuir-Freundlich and Khan, poorly fit the experimental data with low R^2^ values (0.89) for both models. Overall, the graph reveals a sharp increase in adsorption capacity at low concentrations, indicating high affinity between ciprofloxacin and LDH surface, and a plateau at higher concentrations, suggesting saturation of available adsorption sites.

**Table 2:** Isotherm models for adsorption of CIP onto Ni-Al LDH.

Adsorption models		Parameters	Value
**Two- parameter isotherm**	**Langmuir**	q_max_ [mg g^− 1^]	14.03
		K_L_ [L mg^− 1^]	0.191
		R^2^	0.96
	**Freundlich**	K_f_ [L mg^− 1^]	0.677
		1/n_F_	2.34
		R^2^	0.95
	**Dubinin-Radushkevich**	q_max_ [mg g^− 1^]	10.89
		K_ad_ [mol^2^ kJ^− 2^]	0.00046
		R^2^	0.96
**Three- parameter isotherm**	**Langmuir-Freundlich isotherm**	_MLF_ [mg g^−1^]	44.37
		_LF_ [L mg^−1^]	0.020
		_LF_	0.76
		R^2^	0.89
	**Khan**	q_m_ [mg g^− 1^]	1.85
		b_k_	3.4
		a_k_	0.35
		R^2^	0.89
**Four-parameter isotherm**	**Baudu**	q_m_ [mg g^− 1^]	14.39
		b_0_	0.168
		x	0.0
		y	0.0
		R^2^	0.95

### Mechanism of reaction

#### Mechanisms of adsorption

**Fig. 13:**
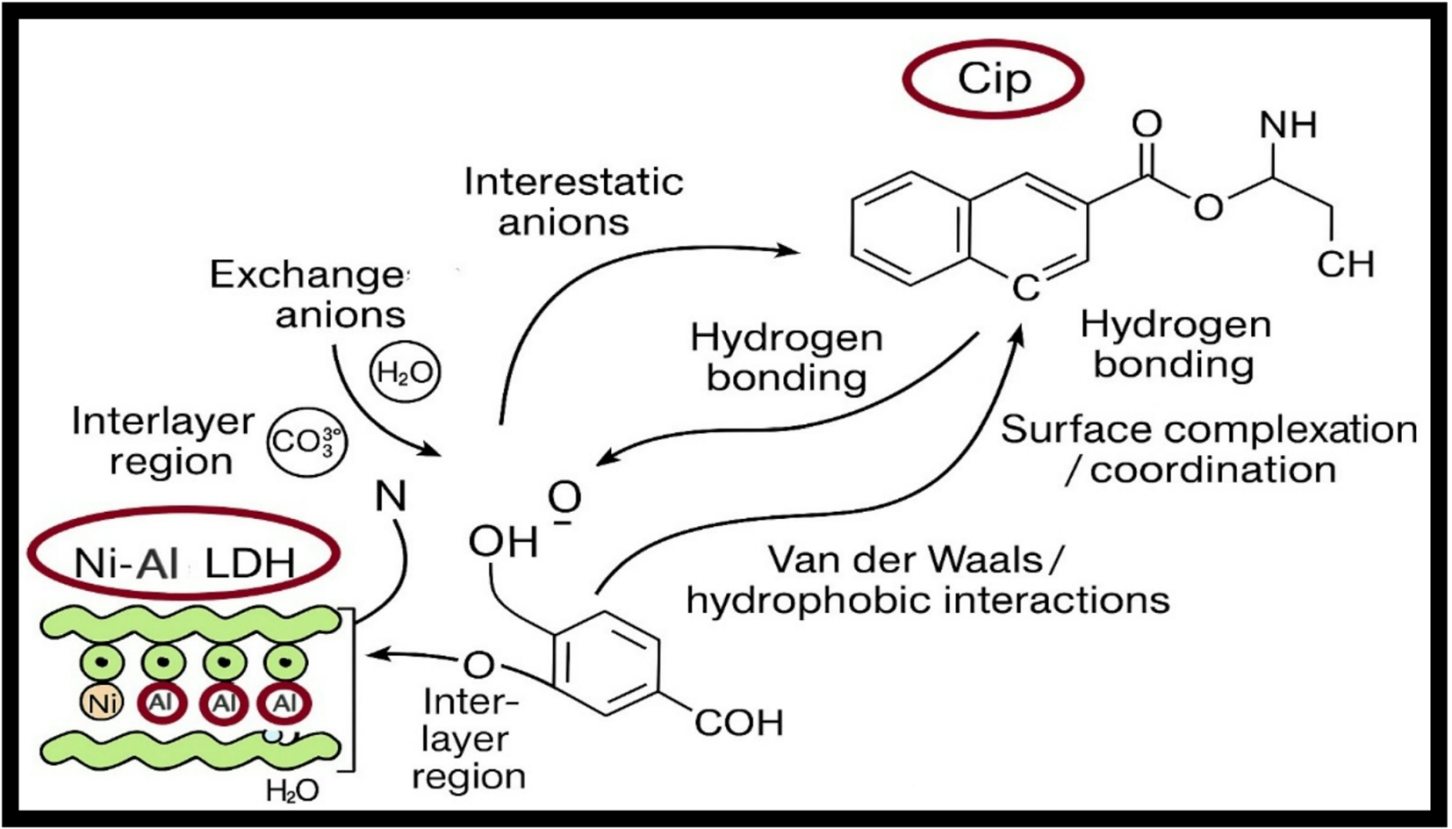
Mechanism of adsorption process.

The adsorption process causes adsorbents to accumulate an outer layer of CIP, or adsorbate, on their surface. adsorption takes place on the solid surface, and the pollutant is carried inside the adsorbent particle. XRD analysis suggests structural alterations, because of CIP molecules intercalating into the LDH layers. The FTIR spectra of Ni-Al LDH before and after CIP adsorption (Figs. 1 and 3) significantly indicated the availability of hydroxyl groups on LDH that may bind to the positively charged CIP through deprotonation. The mechanism of the absorption of CIP onto LDH is described in Figure (13) and could be summed up as following: surface complexation, Hydrogen bonding, ion exchange, Van der Waals/hydrophobic interactions and electrostatic attraction.

Functional groups of organic compounds like ^−^NH, ^−^OH and F-ions act as hydrogen-ponding donors to establish hydrogen bonds with (hydroxyl groups) on the Ni-Al LDH. In surface complexation, the metal centers (Ni²⁺, Al³⁺) may coordinate (form coordination bonds) with functional groups of ciprofloxacin, especially with oxygen or nitrogen donors (like carbonyls, carboxylates, or amines). While in ion exchange, the interlayer region of LDH contains exchangeable anions (like NO₃⁻, CO₃²⁻, etc.). Some of those anions can be exchanged with deprotonated parts of ciprofloxacin (or its entire anionic form).This helps the molecule penetrate the interlayer space in addition to adsorbing on surfaces. Electrostatic attraction happened at pH values where ciprofloxacin is anionic (deprotonated carboxyl groups), the negatively charged parts of the Cipro are attracted to positive sites on LDH layers. The LDH layers (Ni²⁺/Al³⁺ hydroxides) have net positive character(especially when the interlayer anions do not fully compensate) so there’s attraction to negatively charged ciprofloxacin species. Finally, Van der Waals/hydrophobic interactions when parts of the ciprofloxacin molecule that are nonpolar or have aromatic rings may interact via weaker forces (π– π stacking, dispersion forces) with LDH surfaces (especially in less polar regions). These are secondary but contribute to overall adsorption, especially when concentration is high or when other interactions are saturated^72,73^. In the FTIR spectra of the CIP-adsorbed material, the intensity of the OH groups at about 3812.524 cm⁻¹significantly decreases and shifts toward the minimal wavenumbers of 3743.338 cm⁻¹, confirming the presence of dipole-dipole hydrogen bonding.The peak at 3418.681 cm⁻¹ was shifted to 3417.040 cm⁻¹ and it also shows a splitting which may be attributed to the existence of the molecules of CIP with functional groups like, C = O and N–H stretching bonds onto the LDH surface. At 1632.654 cm⁻¹ compared to the original sample (1633.765 cm⁻¹), the H_2_O bending vibrations show a slight decrease, proving that H_2_O molecules are present, and could be displaced or interacted with the CIP molecules. The peak of metal hydroxyl at 1399.225 cm⁻¹, indicates a slight reduction in intensity, even though, the peak is still strong, proving that after the adsorption of CIP molecules, the LDH core is retained. A minor peak formed at 990.513 cm⁻¹ showing the possible interaction among LDH and the adsorbed CIP molecules^43^.

Hence, the mechanism of adsorption includes both physical and chemical interactions. This is agreed with the kinetic modeling of the experimental data where M12O, P1O and P2O can describe this adsorption system.

#### Mechanisms of photocatalytic degradation

**Fig. 14:**
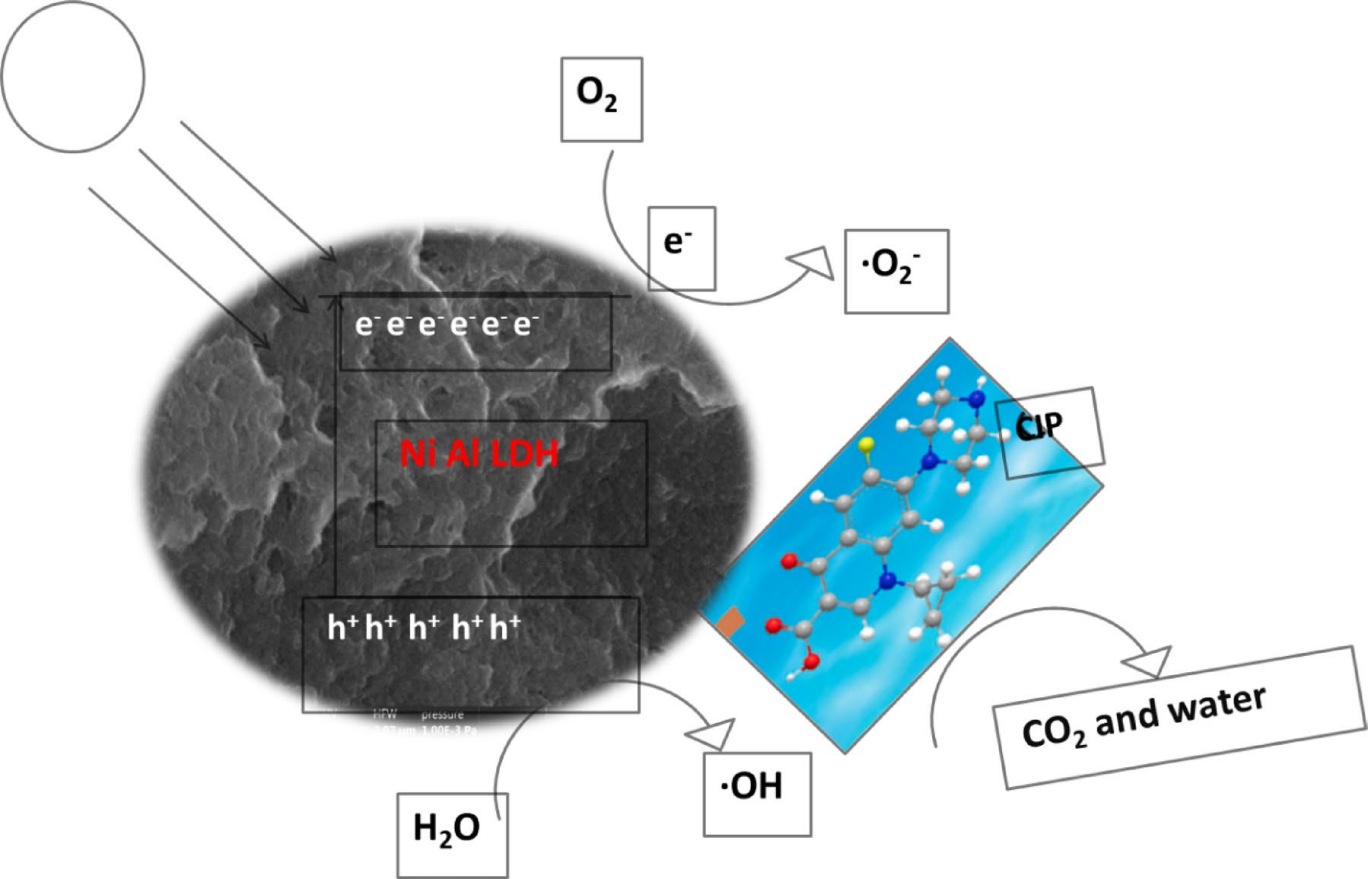
Mechanism of photodegradation process.

Ni- Al DH promote the production of reactive species; superoxide radicals (•O_2_−), hydroxyl radicals (•OH), and notably singlet oxygen (^1^O_2_) from the adsorbed water or/and hydroxyl anion in LDH. As shown in figure (14) these species break down CIP molecules in water. Moreover, the unique composition and structure of the LDH promote the generation and separation of the photogenerated charge carriers, and promote the electron transfer causing the development of ROS that attack and degrade CIP. The main degradation routes includes the hydroxylation at the quinolone core and piperazinyl substituent, cleavage of the piperazine ring, fluorine removal and decarboxylation and oxidative ring opening and bond cleavage by ROS, leading to less toxic byproducts such as CO_2_ and H_2_O^74,75^.

### Current research on the elimination of CIP from water

Table 3 provides a list of recent research on the optimal conditions for the removal of CIP utilizing different catalysts and adsorbents along with the maximum adsorption capacities or/and removal percentages.

**Table 3:** A comparison of the adsorption capacity or removal percentage of different adsorbents and photocatalysts toward CIP.

Adsorbent	pH	Initial conc. (mg L^− 1^)	Contact time (min)	q_max_ (mg g^− 1^) (Removal %)	References
CaMgAl-LDH/Red Mud	7	70	90	138.16	^76^
Regenerated-reed/reed-charcoal	10.42	35	115.28	17.3 (76.66%)	^43^
Bentonite@Pumice	2	10	240	54	^13^
CS/MMT/ZnO hydrogel	7	30	120	56.49	^77^
CoFe-MOF aerogel	7.26	92.42	90	226.8 (64.85%)	^4^
MgAl-LDOs	5	10	420	22.96	^78^
Ni-Al LDH	11	1–50	5	14.38	This study

**Photocatalyst**	**pH**	**Initial conc (**mg L^− 1^**)**	**Contact time (min)**	**Removal percent %**	**References**
Montmorillonite/CuFe_2_O_4_	3	32.5	47.50	83.75	^79^
Graphitic carbon nitride (g-C_3_N_4_) \ Fe_2_O_3_	7	10–50	60	100	^67^
CuPF6/g-CN	8	20	50	98.5	^80^
Bi doped g-C3N		20	60	79	^81^
Na-hydroxyapatite	7	20	120	41.59	^82^
Ni-Al LDH	11	1–50	60	78.7	This study

### Reusability/recyclability

**Fig. 15:**
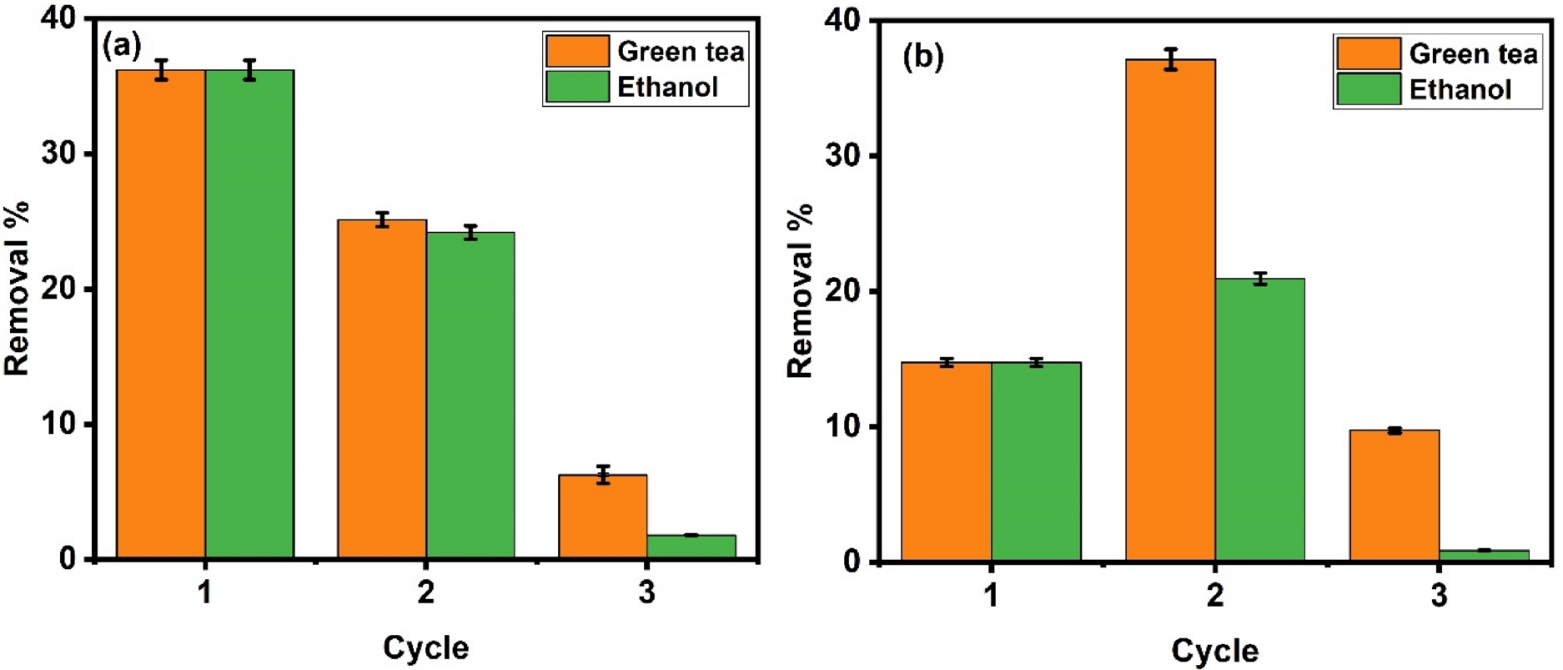
Reusability cycles of Ni-Al LDH after adsorption (**a**) and photodegradation (**b**) using a green solvent (green tea) and a common solvent (ethanol).

The reusability of LDH as an adsorbent and photocatalyst is shown in Fig. 15a and b, respectively. For ethanol, the removal efficiency declined from 36.19% (cycle 1) to 24.2% (cycle 2), this reduction reflects some loss of adsorption capacity. The decrease in efficiency over time suggests a minimal deterioration of the adsorbent. Green tea exhibited initial removal efficiency, achieving 36.19% in the first cycle. However, a reduction was observed in subsequent cycles, with removal efficiencies declining to 25.1% and 6.25% in the second and third cycles, respectively. The observed reduction in removal efficiency for both solvents over successive cycles indicates some loss of adsorption capacity, potentially caused by structural degradation, pore clogging or deactivation of adsorption sites. For the recyclability of the photocatalyst, Fig. 15b shows that in the first cycle, both solvents displayed comparable performance, with removal efficiencies of 14.7%. However, notable differences emerged in subsequent cycles. Green tea showed an initial improvement in efficiency during the second cycle, surpassing ethanol and achieving a peak removal efficiency of 37.1%. This could be attributed to potential enhancement in photocatalytic activity due to organic compounds from green tea acting as co-adsorbents or modifiers. While ethanol achieved a removal efficiency of 20.9% in the second cycle. However, in the third cycle, a sharp decline in efficiency was shown, dropping to 9.7%. This decrease may result from the accumulation of organic residues from green tea, leading to pore blockage or deactivation of active sites on the photocatalyst’s surface. However, green tea demonstrates better consistency and reliability for multiple recycling cycles than methanol for both adsorption and photocatalytic degradation.

### Cost analysis

Table 4 shows the cost breakdown of the Ni-Al LDH. The cost of 1 g of the developed Ni-Al LDH is 12.77 L.E., and this cost is relatively high. However, this cost can be significantly reduced if the cost of drying is reduced. Hence, it is suggested to use solar energy for drying instead of electricity.

**Table 4:** The cost analysis of the prepared Ni-Al LDH.

Material	Quantity unit	Unit price (L.E)	Quantity	Total price (L.E)
NiCl_2_.6H_2_O	g	1.6	9.5	15.2
AlCl_3_	g	1.6	2.66	4.26
The total cost of the materials is 19.45 LE				
Energy	Time (h)	Power max (kW)	Unit cost (L.E.kWh)	Energy cost
Magnetic stirrer	5	1.06	1.28	6.4
Dryer	24	1	2	48
The total cost of energy is 54.4				
The overall cost of 5 g of Ni-Al LDH is 73.85 LE				

### Advantages of the proposed dual approach

Although the synthesized Ni-Al LDH exhibited high efficiency in adsorbing CIP, it is important to recognize that adsorption, as a standalone process, primarily involves the physical or chemical attachment of contaminants onto the surface of the adsorbent. This mechanism effectively reduces the concentration of the pollutant in the liquid phase but does not eliminate it from the system entirely. Instead, it results in the accumulation of pharmaceutical residues on solid material, potentially leading to secondary waste management challenges if the spent adsorbent is not appropriately handled or regenerated. To overcome this limitation and to achieve a more complete and environmentally sustainable remediation process, photocatalysis can be employed as a complementary treatment strategy. The integration of photocatalysis enables the oxidative degradation and mineralization of the adsorbed CIP molecules, converting them into less harmful end products such as carbon dioxide and water. Using Ni-Al LDH as both an efficient adsorbent and a sunlight-responsive photocatalyst enhances the overall treatment efficacy and compensates the limitations of each approach. Moreover, the use of solar energy further contributes to the cost-effectiveness and environmental sustainability of the process. This integrated approach addresses both the removal and degradation of pharmaceutical contaminants.

### Potential industrial applications

The proposed Ni-Al LDH-based method demonstrates high potential for practical application in treating pharmaceutical-contaminated wastewater, particularly in hospital effluents or pharmaceutical industry discharge. This method provides a foundation for the development of water remediation technologies targeting emerging contaminants such as antibiotics.

### Strengths and limitations of the study

The developed Ni-Al LDH serves both as an adsorbent and a photocatalyst for removing CIP. Furthermore, the use of solar irradiation instead of artificial UV light supports environmentally friendly, low-energy operation. Throughout this study, it was assumed that the reaction conditions, such as temperature and pH, remained constant during the experiments, and that the systems were well-mixed and stirred, ensuring unified exposure to light. The kinetic and isothermal modelling applied assume ideal conditions with no interference from competing compounds, which may not reflect the complexity of real wastewater matrices. However, the approach has many advantages; study has notable limitations. Based on the regeneration results, the developed Ni-Al LDH exhibits limited reusability, with a significant decline in performance after only two cycles, limiting its practical application on real wastewater. Further studies are required to enhance its stability. Additionally, the synthesis of the LDH requires costly precursors and conditions, making it challenging for large-scale or industrial implementation. Although the adsorption capacity and photocatalytic degradation are promising, the removal efficiency still needs further enhancement to meet the efficiency requirements of actual wastewater treatment. Future research should therefore focus on increasing the removal performance, modifying the LDH structure to improve recyclability, identifying more cost-effective synthesis methods, testing under real environmental conditions, and incorporating the effect of coexisting substances typically found in wastewater.

## Conclusion

This research demonstrates the effectiveness of Ni-Al LDH as a promising material for the remediation of CIP, through adsorption and photocatalytic degradation processes. With a molar ratio of (Ni-Al) 4:1, Ni-Al LDH was produced via the technique of co-precipitation. The samples were characterized before and after the adsorption and the photocatalytic degradation, applying FESEM/EDX, XRD, FTIR, XPS, TGA, and BET analyses. Adsorption and photocatalytic degradation processes were affected by many factors. However, the optimum parameters for the adsorption and photocatalytic degradation were pH 11, doses 0.02 and 0.125 g, and contact time 5 and 60 min, respectively. For the kinetic models, the M12O model was found to be the best fitted model. For the adsorption isothermal models, however, Langmuir model provided the best fit. The material reached a q_max_ of 14.03 mg g^− 1^. These results underscore the potential of Ni-Al LDH as a dual-functional, sustainable solution for removing drug residues from water, addressing environmental pollution, and contributing to safer water resources.

## Supplementary Information

Below is the link to the electronic supplementary material.

Supplementary Material 1

## Data Availability

All data listed or discussed during this work are included in this published article.

## References

[1] Rao, J. N. & Parsai, T. Pollution and toxicity of heavy metals in wildfires-affected soil and surface water: A review and meta-analysis. *Environ. Pollut*. **369**, 125845 (2025). 39954764 10.1016/j.envpol.2025.125845

[2] Patel, P. K. & Uppaluri, R. V. S. Environmental sustainability through adsorption: A review of chitosan’s potential in dye pollution remediation. *Sustain. Chem. Pharm.* **46**, 102096 (2025).

[3] Akhil, D., Lakshmi, D., Senthil Kumar, P., Vo, D. V. N. & Kartik, A. Occurrence and removal of antibiotics from industrial wastewater. *Environ. Chem. Lett.* **19**, 1477–1507 (2021).

[4] Kim, N., Tran, T., Tran, C., Thanh Ngan Tran, A. & Nhan Le, T. H. Tan Lam, V. Optimization of Ciprofloxacin adsorption onto CoFe-MOF aerogel cylinders based on response surface methodology: adsorption kinetics, isotherm models. *Mater. Sci. Eng. B*. **297**, 116694 (2023).

[5] Soltani, R., Pelalak, R., Pishnamazi, M., Marjani, A. & Shirazian, S. A water-stable functionalized NiCo-LDH/MOF nanocomposite: green synthesis, characterization, and its environmental application for heavy metals adsorption. *Arab. J. Chem.* **14**, 103052 (2021).

[6] Larsson, D. G. J. & Flach, C. F. Antibiotic resistance in the environment. *Nat. Rev. Microbiol.* **20**, 257–269 (2022). 34737424 10.1038/s41579-021-00649-x PMC8567979

[7] Lin, B. et al. Enhanced peroxymonosulfate activation by Co/Mn and P modified carbon nitride for Ciprofloxacin degradation: Performance, mechanism and toxicity assessment. *J. Environ. Chem. Eng.* **13**, 116604 (2025).

[8] Zhang, T. et al. Study on Preparation of a pizza-like attapulgite-based composite membrane and its performance on methylene blue and Ciprofloxacin removal. *Sep. Purif. Technol.* **359**, 130521 (2025).

[9] Fang, N. et al. Insight into adsorption kinetics, equilibrium, thermodynamics, and modeling of Ciprofloxacin onto iron ore tailings. *Water* **17**, 760 (2025).

[10] Meng, L. et al. Mutual enhancement of metal coagulant and peroxymonosulfate for Ciprofloxacin removing and the multiple-mode mechanisms. *Sep. Purif. Technol.* **320**, 124152 (2023).

[11] Wang, M., Xu, Z., Huang, Y. & Dong, B. Biodegradation of Ciprofloxacin by a manganese-oxidizing fungus cladosporium sp. XM01: performance and transcriptome analysis. *J. Hazard. Mater.* **494**, 138543 (2025). 40344838 10.1016/j.jhazmat.2025.138543

[12] Idrees, M. et al. Advancements in photocatalytic systems for Ciprofloxacin degradation, efficiency, mechanisms, and environmental considerations. *J. Mol. Liq*. **424**, 127115 (2025).

[13] Khan, A. H., Aziz, H. A., Palaniandy, P. & Zouli, N. Ciprofloxacin adsorption onto Pumice-bentonite composites: Modeling, kinetics, equilibriums and reusability studies. *J. Taiwan. Inst. Chem. Eng.* **166**, 105618 (2025).

[14] Elkomy, A. S., Abdel-wahab, S., Shehata, N. & M. & A comparison between adsorption and photocatalytic degradation for the management of sulfamethoxazole in water. *Sci. Rep.* **15**, 13576 (2025). 40253419 10.1038/s41598-025-95947-2 PMC12009432

[15] Xu, M. et al. Performance enhancement strategies of bi-based photocatalysts: A review on recent progress. *Chem. Eng. J.* **389**, 124402 (2020).

[16] Cheikh, S. et al. Complete Elimination of the Ciprofloxacin Antibiotic from Water by the Combination of Adsorption–Photocatalysis Process Using Natural Hydroxyapatite and TiO2. *Catalysts.* **13** (2023). 10.3390/catal13020336

[17] Wang, J., Pi, H., Zhao, P. & Zhou, N. Efficient removal of Methyl orange and Ciprofloxacin by reusable Eu–TiO 2/PVDF membranes with adsorption and photocatalysis methods. *RSC Adv.* **14**, 18432–18443 (2024). 38860257 10.1039/d4ra02962c PMC11163413

[18] Mahmoud, R. et al. Investigation of ternary Zn–Co–Fe layered double hydroxide as a multifunctional 2D layered adsorbent for Moxifloxacin and antifungal disinfection. *Sci. Rep.* **14**, 806 (2024). 38191628 10.1038/s41598-023-48382-0 PMC10774404

[19] Selvaraj, V. & Karuppasamy, G. Layered double hydroxide nanocomposites: a promising platform for sustainable photocatalytic solutions—a short review. *J. Nanoparticle Res.* **27**, 1–33 (2025).

[20] Bobde, P. et al. Layered double hydroxides (LDHs)-based photocatalysts for dye degradation: A review. *Int. J. Environ. Sci. Technol.* **20**, 5733–5752 (2023).

[21] Xie, M. et al. A review of the degradation of antibiotic contaminants using advanced oxidation processes: modification and application of layered double hydroxides based materials. *Environ. Sci. Pollut Res.* **31**, 18362–18378 (2024). 10.1007/s11356-024-32059-w 38353817

[22] Zaher, A., Taha, M., Farghali, A. A. & Mahmoud, R. K. Zn/Fe LDH as a clay-like adsorbent for the removal of Oxytetracycline from water: combining experimental results and molecular simulations to understand the removal mechanism. *Environ. Sci. Pollut Res.* **27**, 12256–12269 (2020). 10.1007/s11356-020-07750-3 31993907

[23] Zaher, A., Taha, M. & Mahmoud, R. K. Possible adsorption mechanisms of the removal of Tetracycline from water by La-doped Zn-Fe-layered double hydroxide. *J Mol. Liq* **322**, 114546 10.1016/j.molliq.2020.114546 (2021).

[24] Li, X., Shi, X. L., Wang, J., Shi, K. & Wang, Q. Effect of different hydrogen donors on the catalytic conversion of levulinic acid to γ-valerolactone over non-noble metal catalysts. *J. Ind. Eng. Chem.* **138**, 17–33 (2024).

[25] Fang, Q., Sun, Q., Zhong, R., Wang, H. & Qi, J. Recent advances in doping engineering of heterogeneous catalyst for carbon dioxide hydrogenation. *Mater. Today Chem.* **46**, 102770 (2025).

[26] Monash, P. & Pugazhenthi, G. Utilization of calcined Ni-Al layered double hydroxide (LDH) as an adsorbent for removal of Methyl orange dye from aqueous solution. *Environ. Prog Sustain. Energy*. **33**, 154–159 (2014).

[27] Lagergren, S. Zur theorie der sogenannten adsorption geloster Stoffe. *K Sven Vetenskapsakademiens Handl.* **24**, 1–39 (1898).

[28] Ho, Y. S. & McKay, G. Pseudo-second order model for sorption processes. *Process. Biochem.* **34**, 451–465 (1999).

[29] Avrami, M. Kinetics of phase change. I general theory. *J. Chem. Phys.* **7**, 1103–1112 (1939).

[30] Lopes, E. C. N., dos Anjos, F. S. C., Vieira, E. F. S. & Cestari, A. R. An alternative avrami equation to evaluate kinetic parameters of the interaction of hg (II) with thin Chitosan membranes. *J. Colloid Interface Sci.* **263**, 542–547 (2003). 12909046 10.1016/s0021-9797(03)00326-6

[31] Weber Jr, W. J. & Morris, J. C. Kinetics of adsorption on carbon from solution. *J. Sanit. Eng. Div.* **89**, 31–59 (1963).

[32] Irani, M., Mohammadi, T. & Mohebbi, S. Photocatalytic degradation of methylene blue with ZnO nanoparticles; a joint experimental and theoretical study. *J. Mex Chem. Soc.* **60**, 218–225 (2016).

[33] Langmuir, I. The adsorption of gases on plane surfaces of glass, mica and platinum. *J. Am. Chem. Soc.* **40**, 1361–1403 (1918).

[34] Freundlich, H. Über die adsorption in lösungen. *Z. für Phys. Chemie*. **57**, 385–470 (1907).

[35] Dubinin, M. M. The equation of the characteristic curve of activated charcoal. *Dokl. Akad. Nauk. SSSR*. **55**, 327–329 (1947).

[36] Brouers, F. & Sotolongo-Costa, O. Generalized fractal kinetics in complex systems (application to biophysics and biotechnology). *Phys. Stat. Mech. Its Appl.* **368**, 165–175 (2006).

[37] Khan, A. A. & Singh, R. P. Adsorption thermodynamics of Carbofuran on Sn (IV) arsenosilicate in H+, Na + and Ca2 + forms. *Colloids Surf.* **24**, 33–42 (1987).

[38] Fu, Y. et al. Chloride intercalated Ni-Al layered double hydroxide for effective adsorption removal of Sb(Ⅴ). *Inorg. Chem. Commun.* **142**, 109651 (2022).

[39] Ghanbari, N. & Ghafuri, H. Design and Preparation the novel polymeric layered double hydroxide nanocomposite (LDH/Polymer) as an efficient and recyclable adsorbent for the removal of methylene blue dye from water. *Environ. Technol. Innov.* **26**, 102377 (2022).

[40] Feng, Y., Li, D., Wang, Y., Evans, D. G. & Duan, X. Synthesis and characterization of a UV absorbent-intercalated Zn–Al layered double hydroxide. *Polym. Degrad. Stab.* **91**, 789–794 (2006).

[41] Guo, Y., Zhu, Z., Qiu, Y. & Zhao, J. Adsorption of arsenate on Cu/Mg/Fe/La layered double hydroxide from aqueous solutions. *J. Hazard. Mater.* **239**, 279–288 (2012). 23000241 10.1016/j.jhazmat.2012.08.075

[42] Charafi, S. et al. Adsorption and reusability performances of Ni/Al layered double hydroxide for the removal of eriochrome black T dye. *Biointerface Res. Appl. Chem* **13**(3), 265–271 (2023).

[43] Karoui, S. et al. Synthesis of novel biocomposite powder for simultaneous removal of hazardous Ciprofloxacin and methylene blue: central composite design, kinetic and isotherm studies using Brouers-Sotolongo family models. *J. Hazard. Mater.* **387**, 121675 (2020). 31767503 10.1016/j.jhazmat.2019.121675

[44] Shabanian, M., Hajibeygi, M. & Raeisi, A. 2 - FTIR characterization of layered double hydroxides and modified layered double hydroxides. in *Woodhead Publishing Series in Composites Science and Engineering* (eds. Thomas, S. & Daniel, S. B. T.-L. D. H. P. N.) 77–101, Woodhead Publishing, (2020). 10.1016/B978-0-08-101903-0.00002-7

[45] Waheed, T. et al. Enhanced photocatalytic performance of Cr/Ni/Mg/Al layered double hydroxides against Methyl orange in aqueous solution. *Desalin. Water Treat.* **319**, 100495 (2024).

[46] Rives, V. & Ulibarri, M. A. Layered double hydroxides (LDH) intercalated with metal coordination compounds and oxometalates. *Coord. Chem. Rev.* **181**, 61–120 (1999).

[47] Li, X. et al. Hierarchical NiAl LDH nanotubes constructed via atomic layer deposition assisted method for high performance supercapacitors. *Electrochim. Acta*. **255**, 15–22 (2017).

[48] Zhan, T., Zhang, Y., Liu, X., Lu, S. & Hou, W. NiFe layered double hydroxide/reduced graphene oxide nanohybrid as an efficient bifunctional electrocatalyst for oxygen evolution and reduction reactions. *J. Power Sources*. **333**, 53–60 (2016).

[49] Zhan, T., Tan, Z., Wang, X. & Hou, W. Hemoglobin immobilized in g-C3N4 nanoparticle decorated 3D graphene-LDH network: direct electrochemistry and electrocatalysis to trichloroacetic acid. *Sens. Actuators B Chem.* **255**, 149–158 (2018).

[50] Liang, J. et al. Constructing high-efficiency photocatalyst for degrading ciprofloxacin: Three-dimensional visible light driven graphene based NiAlFe LDH. *J. Colloid Interface Sci.* **540**, 237–246 (2019). 30641401 10.1016/j.jcis.2019.01.011

[51] Gupta, N. K., Saifuddin, M., Kim, S. & Kim, K. S. Microscopic, spectroscopic, and experimental approach towards Understanding the phosphate adsorption onto Zn–Fe layered double hydroxide. *J. Mol. Liq*. **297**, 111935 (2020).

[52] Lu, Z. H. et al. Synthesis and adsorption properties investigation of Fe3O4@ZnAl-LDH@MIL-53(Al) for Azole fungicides removal from environmental water. *Sep. Purif. Technol.* **276**, 119282 (2021).

[53] Feng, G. et al. Preparation of novel porous hydroxyapatite sheets with high Pb2 + adsorption properties by self-assembly non-aqueous precipitation method. *Ceram. Int.* **49**, 30603–30612 (2023).

[54] Ahmed, I. M., Abd-Elhamid, A. I., Aly, A. A., Bräse, S. & Nayl, A. A. Synthesis of Ni-Fe-CO3 layered double hydroxide as effective adsorbent to remove Cr(VI) and ARS-dye from aqueous media. *Environ. Technol. Innov.* **31**, 103214 (2023).

[55] Machrouhi, A. et al. Experimental and density functional theory studies of Methyl orange adsorption on Ni-Al/LDH intercalated sodium Dodecyl sulfate. *Chem. Phys. Impact*. **6**, 100214 (2023).

[56] Ghafuri, H. & Ghanbari, N. Design and synthesis of LDH nano composite functionalized with trimesic acid and its environmental application in adsorbing organic dyes indigo carmine and methylene blue. *Heliyon.* **10**, (2024). 10.1016/j.heliyon.2024.e33656 PMC11295991 39100470

[57] Altalhi, A. A., Mohamed, E. A. & Negm, N. A. Recent advances in layered double hydroxides (LDH)-based materials: Fabrications, modification strategies, characterization, promising environmental catalytic applications, and prospective aspects. *Energy Adv* **3**, 2136–2151 10.1039/D4YA00272E(2024).

[58] Janani, F. Z. et al. Effect of ag doping on photocatalytic activity of ZnO-Al2O3 derived from LDH structure: Synthesis, characterization and experimental study. *Appl. Surf. Sci. Adv.* **16**, 100430 (2023).

[59] Duddi, R. et al. Hydrothermally synthesised porous NiAl-LDH as an efficient pseudocapacitive material in asymmetric supercapacitors. *Hybrid. Adv.* **8**, 100372 (2025).

[60] Wijaya, A. et al. Biohybrid ZnCr-LDH/Spirogyra algae composite for selective adsorption from multi-dye anionic mixtures: toward enhanced removal of direct yellow. *Next Mater.* **9**, 101094 (2025).

[61] Jefri, J. et al. Boosting photocatalytic efficiency: LDH modified with gambier leaf extract for Tetracycline degradation. *Next Mater.* **7**, 100633 (2025).

[62] Li, S., Huang, T., Du, P., Liu, W. & Hu, J. Photocatalytic transformation fate and toxicity of Ciprofloxacin related to dissociation species: experimental and theoretical evidences. *Water Res.* **185**, 116286 (2020). 32818732 10.1016/j.watres.2020.116286

[63] Qiu, S. et al. Investigation of protonation and deprotonation processes of kaolinite and its effect on the adsorption stability of rare Earth elements. *Colloids Surf. Physicochem Eng. Asp*. **642**, 128596 (2022).

[64] da Silva Bruckmann, F. et al. Adsorption of Ciprofloxacin from aqueous solution and fresh synthetic urine by graphene oxide: conventional and statistical physics modeling approaches. *Chem. Eng. J.* **487**, 150484 (2024).

[65] Karunakaran, K., Usman, M. & Sillanpää, M. A. Review on Superadsorbents with Adsorption Capacity ≥ 1000 mg g – 1 and Perspectives on Their Upscaling for Water/Wastewater Treatment. *Sustainability* . **14** (2022). 10.3390/su142416927

[66] Badawi, A. K., Abd Elkodous, M. & Ali, G. A. M. Recent advances in dye and metal ion removal using efficient adsorbents and novel nano-based materials: an overview. *RSC Adv.* **11**, 36528–36553 (2021). 35494372 10.1039/d1ra06892j PMC9043615

[67] Rajiv, P., Mengelizadeh, N., McKay, G. & Balarak, D. Photocatalytic degradation of Ciprofloxacin with Fe2O3 nanoparticles loaded on graphitic carbon nitride: mineralisation, degradation mechanism and toxicity assessment. *Int. J. Environ. Anal. Chem.* **103**, 2193–2207 (2023).

[68] Tran, H. N., You, S. J., Hosseini-Bandegharaei, A. & Chao, H. P. Mistakes and inconsistencies regarding adsorption of contaminants from aqueous solutions: A critical review. *Water Res.* **120**, 88–116 (2017). 28478298 10.1016/j.watres.2017.04.014

[69] Chong, M. N., Jin, B., Chow, C. W. K. & Saint, C. Recent developments in photocatalytic water treatment technology: A review. *Water Res.* **44**, 2997–3027 (2010). 20378145 10.1016/j.watres.2010.02.039

[70] Haleem, A., Shafiq, A., Chen, S. Q. & Nazar, M. A. Comprehensive Review on Adsorption, Photocatalytic and Chemical Degradation of Dyes and Nitro-Compounds over Different Kinds of Porous and Composite Materials. *Molecules.* **28, **(2023). 10.3390/molecules28031081 10.3390/molecules28031081 PMC9918932 36770748

[71] Kostić, M. et al. Ultrasound-assisted synthesis of a new material based on MgCoAl-LDH: characterization and optimization of sorption for progressive treatment of water. *Environ. Technol. Innov.* **26**, 102358 (2022).

[72] Ji, L., Chen, W., Duan, L. & Zhu, D. Mechanisms for strong adsorption of Tetracycline to carbon nanotubes: a comparative study using activated carbon and graphite as adsorbents. *Environ. Sci. Technol.* **43**, 2322–2327 (2009). 19452881 10.1021/es803268b

[73] Zeng, Z. et al. Comprehensive adsorption studies of Doxycycline and Ciprofloxacin antibiotics by biochars prepared at different temperatures. *Front. Chem.* **6**, 80 (2018). 29637067 10.3389/fchem.2018.00080 PMC5880934

[74] Sahoo, S., Behera, A., Mansingh, S., Tripathy, B. & Parida, K. Facile construction of CoWO4 modified g-C3N4 nanocomposites with enhanced photocatalytic activity under visible light irradiation. *Mater. Today Proc.* **35**, 193–197 (2021).

[75] Nivetha, M. S., Kumar, J. V., Arulmozhi, R. & Abirami, N. Ultrathin 2D type-II heterojunctions ZCLDH/CN with a higher photocatalytic performance of Ciprofloxacin under simulated light illumination. *Opt. Mater. (Amst)*. **140**, 113861 (2023).

[76] Ali Hammood, Z. & Mohammed, A. Enhanced adsorption of Ciprofloxacin from an aqueous solution using a novel CaMgAl-layered double hydroxide/red mud composite. *Results Eng.* **23**, 102600 (2024).

[77] Sayyar, Z., Hosseini, Z., Mohammadzadeh Pakdel, P. & Hassani, A. Preparation of novel and low-cost Chitosan modified with montmorillonite/ZnO hydrogel nanocomposite for adsorption of Ciprofloxacin from water. *J. Water Process. Eng.* **63**, 105449 (2024).

[78] Gao, R. et al. Preparation of MgAl layered double oxides and its adsorption kinetics for Ciprofloxacin hydrochloride in water. *Environ. Prog Sustain. Energy*. **41**, e13899 (2022).

[79] Al-Musawi, T. J., Mengelizadeh, N., Alwared, A. I., Balarak, D. & Sabaghi, R. Photocatalytic degradation of Ciprofloxacin by MMT/CuFe2O4 nanocomposite: characteristics, response surface methodology, and toxicity analyses. *Environ. Sci. Pollut Res.* **30**, 70076–70093 (2023). 10.1007/s11356-023-27277-7 37145364

[80] Nejat, R. Enhancing the photocatalytic efficiency of g-C_3_N_4_ for ciprofloxacin degradation using Tetrakis (acetonitrile) copper(I) hexafluorophosphate as a highly effective cocatalyst. *Heliyon.* **10**, (2024). 10.1016/j.heliyon.2024.e35829 PMC11382030 39253175

[81] Saini, P. et al. Bismuth doped g-C3N4 composites for enhanced photocatalytic degradation of Ciprofloxacin. *J. Mol. Struct.* **1321**, 140013 (2025).

[82] Tarannum, S., Hossain, M. S., Bashar, M. S., Bahadur, N. M. & Ahmed, S. Amplification of photocatalytic degradation of antibiotics (amoxicillin, ciprofloxacin) by sodium doping in nano-crystallite hydroxyapatite. *RSC Adv.* **14**, 12386–12396 (2024). 38638810 10.1039/d4ra00126e PMC11025524

