# Supplementary figures and images for: Optimization and kinetic modeling of ciprofloxacin adsorption and photocatalytic degradation in water
Source: Sci Rep. 2025 Dec 8;15:43370. doi: 10.1038/s41598-025-29266-x (PMC12690150; doi:10.1038/s41598-025-29266-x)

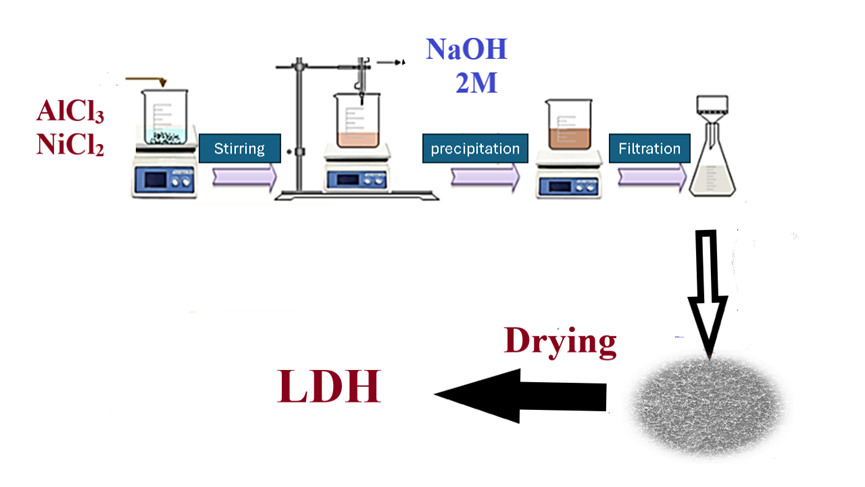


Fig S1 : A schematic diagram illustrating the synthesis

Supplement: Supplementary file 1 — Supplementary Material 1 [file 41598_2025_29266_MOESM1_ESM.docx]
